# Three‐Channel Electron Engineered Fe‐88A@CeO_2_/CDs Nanozyme with Enhanced Oxidase‐Like Activity for Efficient Biomimetic Catalysis

**DOI:** 10.1002/advs.202509713

**Published:** 2025-08-31

**Authors:** Kai Liu, Haibing Zhu, Feng Shi, Juan Li, Xiang Li, Zijun Lai, Haibo Li, Hao Zeng, Zhanjun Yang, Huan Pang

**Affiliations:** ^1^ School of Chemistry and Chemical Engineering Yangzhou University Yangzhou 225002 P.R. China; ^2^ National Engineering Research Center of Immunological Department of Microbiology and Biochemical Pharmacy College of Pharmacy and Laboratory Medicine Third Military Medical University Chongqing 400037 P.R. China

**Keywords:** bimodal immunoassay, biomimetic catalysis, channel‐electron engineering, MOF Nanozyme, oxidase‐like activity

## Abstract

Iron‐based metal‐organic framework (MOF) nanozymes have garnered considerable attention owing to a large specific surface area, adjustable porosity, large Fe‐O clusters, and unsaturated Fe sites. However, the sluggish charge‐transfer rate and restricted active sites of the nanozymes lead to poor enzyme‐like activity and further impede their biomimetic catalysis. Herein, a three‐channel electron‐engineered Fe‐88A@CeO_2_/carbon dots (Fe‐88A@CeO_2_/CDs) nanozyme is proposed for efficient biomimetic catalysis. Fe‐88A@CeO_2_/CDs nanozyme is prepared by incorporation of CeO_2_ and CDs into the porosity of Fe‐88A. Specifically, the original Fe (II)/Fe (III) and the introduced Ce (III)/Ce (IV) redox couples of the nanozyme constitute a dual electron transfer channel. Furthermore, the presence of CDs produces another electron transfer channel. The three‐channel electron engineering strategy for nanozymes can accelerate the electron transfer process accompanied with more active sites, thereby greatly enhancing the oxidase‐like activity of Fe‐88A@CeO_2_/CDs for biomimetic catalysis. The nanozyme can efficiently convert oxygen to ·$O_{2}^{-}$, oxidizing colorless 3,3′,5,5′‐tetramethylbenzidine (TMB) to blue ox‐TMB, and meanwhile the ox‐TMB effectively quenches the fluorescence of CDs. As proof of concept, the nanozyme is utilized to construct a colorimetric‐fluorescence bimodal immunosensor for monitoring Staphylococcal enterotoxin B with excellent performance. This work provides promising insight into designing excellent nanozymes for effective biomimetic catalysis in various fields.

## Graphical abstract

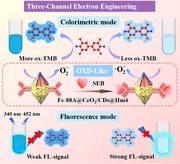

## 1 Introduction

Staphylococcus enterotoxin B (SEB) poisoning poses a significant threat to public health and safety.^[^ ^1^, ^2^ ^]^ Consequently, the rapid and accurate identification and detection of SEB are of paramount importance. In recent years, immunoassays have emerged as the primary method for SEB detection, encompassing techniques such as electrochemistry,^[^ ^3^ ^]^ colorimetry,^[^ ^4^ ^]^ fluorescence,^[^ ^5^ ^]^ and chemiluminescence.^[^ ^6^ ^]^ Among these methods, colorimetric and fluorescent immunosensors are widely utilized due to the advantages of visualization, rapidity, and simple manipulation.^[^ ^7^, ^8^, ^9^ ^]^ However, these two methods usually require natural enzymes or fluorescent dyes to label trace antibodies. It is well‐known that natural enzymes are high‐cost and susceptible to inactivation,^[^ ^10^, ^11^, ^12^, ^13^ ^]^ and fluorescent dyes suffer from photobleaching and blinking,^[^ ^14^, ^15^, ^16^ ^]^ which significantly restricts their practical applications. Meanwhile, current single‐mode detection of SEB is susceptible to interference and exhibits low accuracy.^[^ ^17^ ^]^ As enzyme‐like nanomaterials, nanozymes have gained great attention nowadays due to their high stability, low‐cost, and adjustable catalytic activity.^[^ ^18^, ^19^, ^20^ ^]^ Especially, nanozyme‐based two‐mode biosensors have become more and more popular in biosensing fields.^[^ ^21^ ^]^ Therefore, it is very essential to construct powerful nanozymes with high enzyme‐like activity for biomimetic catalysis and fluorescence properties for the development of bimodal immunoassay to achieve sensitive and accurate detection of SEB.

Iron‐based metal–organic framework (MOF) nanozymes are preferred by researchers on account of their large specific surface area, adjustable porosity, large Fe─O clusters, and unsaturated Fe sites.^[^ ^22^, ^23^, ^24^ ^]^ However, traditional Fe‐88A shows poor enzyme‐like activity due to the drawbacks such as the sluggish charge‐transfer rate and the restricted active sites, which constrains its potential application in biomimetic catalysis.^[^ ^25^, ^26^ ^]^ Therefore, it is highly necessary to improve the active sites and charge‐transfer rate of Fe‐88A to enhance its enzyme‐like activity. Recently, the incorporation of certain semiconductors or carbon materials into MOFs has been extensively investigated in electrocatalysis and battery applications.^[^ ^27^, ^28^ ^]^ In virtue of this strategy, the electrocatalysis activity of MOFs was greatly improved due to the more electronic channels and active sites for accelerating electron transfer.^[^ ^29^, ^30^ ^]^ As a wide‐bandgap semiconductor, CeO_2_ possesses a unique electronic structure that not only modulates the electron density of atoms but also enhances the activity and stability of composite materials.^[^ ^31^, ^32^ ^]^ Additionally, CeO_2_ exhibits multi‐enzyme‐like activities due to the reversible interconversion between Ce^3+^ and Ce^4+^, which has garnered significant attention in sensing applications.^[^ ^33^ ^]^ Carbon dots (CDs) are an emerging class of carbon nanomaterials that exhibit strong fluorescence properties and affinity as well as additional active sites for accelerating rapid electron transfer of composites.^[^ ^34^, ^35^ ^]^ These composites have achieved notable success in photocatalysis, electrochemical catalysis, and drug delivery.^[^ ^36^, ^37^ ^]^ Although researches demonstrate that the CeO_2_ and CDs have been separately utilized to regulate the enzyme‐like activity of MOF‐based nanozymes, very few reports are related to exploring the channel electronic mechanism for enhancing enzyme‐like activity.^[^ ^38^, ^39^, ^40^, ^47^ ^]^ Currently, multi‐channel electronic strategies have been predominantly exploited to enhance the photocatalysis and electrocatalysis properties of MOF‐based catalysts.^[^ ^41^, ^42^, ^43^ ^]^ As far as we know, there are no reports related to the multi‐channel electron strategy for regulating the enzyme‐like activity of MOF nanozymes.^[^ ^44^, ^45^ ^]^ Therefore, the integration of CeO_2_ and CDs into the Fe‐88A nanozyme to enhance catalytic performance through multi‐channel electronic strategy holds significant scientific and practical value.

**Scheme 1:**
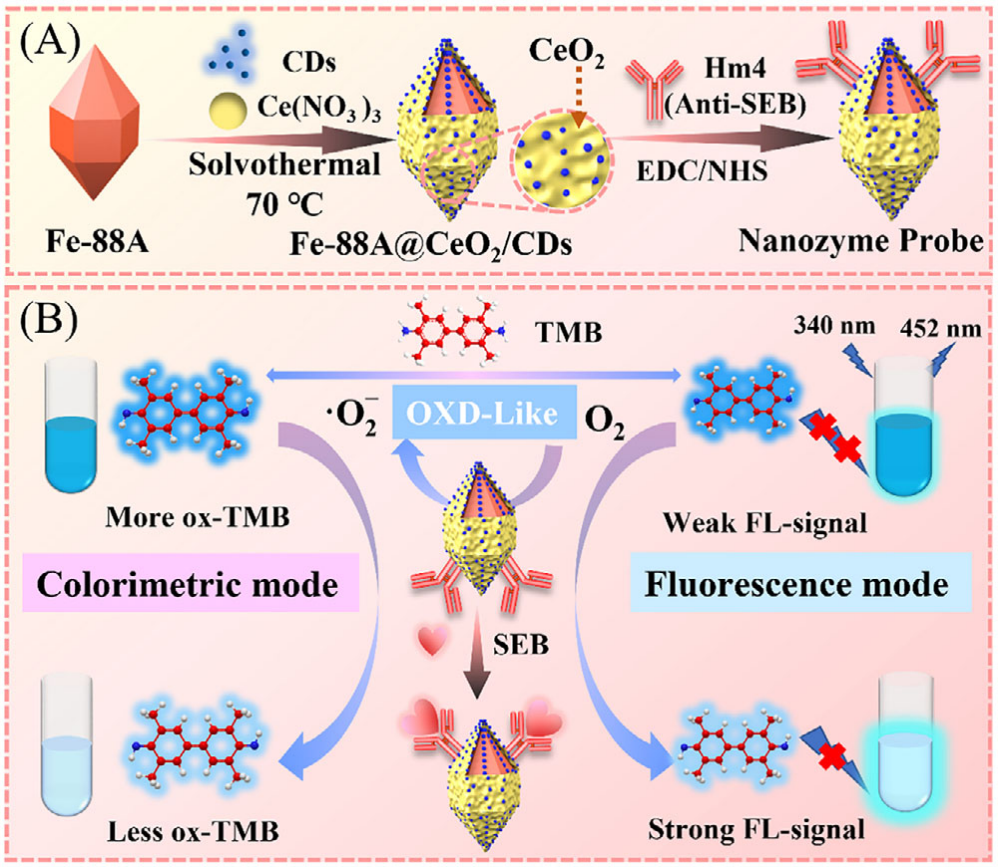
A) Schematic illustration for synthesizing Fe‐88A@CeO_2_/CDs nanozyme and Fe‐88A@CeO_2_/CDs@Hm4 probe; B) Detection process of label‐free colorimetric‐fluorescence bimodal immunoassay for SEB.

Herein, we developed a three‐channel electron engineering strategy to construct a versatile Fe‐88A@CeO_2_/carbon dots (Fe‐88A@CeO_2_/CDs) nanozyme with enhanced oxidase‐like activity for efficient biomimetic catalysis. As depicted in **Scheme** **1**, Fe‐88A@CeO_2_/CDs nanozyme was fabricated by incorporation of CeO_2_ and CDs into the porosity of Fe‐88A. The presence of CeO_2_ and CDs in Fe‐88A endows the composite with three‐channel electron and fluorescence property. This feature improves the bandgap and active sites of the composite and induces the acceleration of electron transfer, thereby greatly enhancing oxidase (OXD)‐like activity of Fe‐88A@CeO_2_/CDs in the process of biomimetic catalysis. The nanozyme is capable of catalyzing oxygen to ·$O_{2}^{-}$ that further oxidizes the colorless 3,3′,5,5′‐tetramethylbenzidine (TMB) to blue ox‐TMB. Meanwhile, the generated ox‐TMB effectively quenches the fluorescence of Fe‐88A@CeO_2_/CDs through the inner filter effect (IFE). The Fe‐88A@CeO_2_/CDs‐based bimodal immunosensor was established by coupling the SEB antibody (Hm4) to Fe‐88A@CeO_2_/CDs. The specific recognition of SEB by immunosensor can inhibit the OXD‐like activity of Fe‐88A@CeO_2_/CDs, leading to a decrease in colorimetric signals and the increase in fluorescence signals. The Fe‐88A@CeO_2_/CDs@Hm4‐based bimodal immunosensor can be successfully used for label‐free colorimetric‐fluorescence bimodal detection of SEB, and holds significant potential in food safety and water safety management.

## 2 Results and Discussion

### 2.1 Characterization of Fe‐88A@CeO_2_/CDs Nanozyme

**Figure 1:**
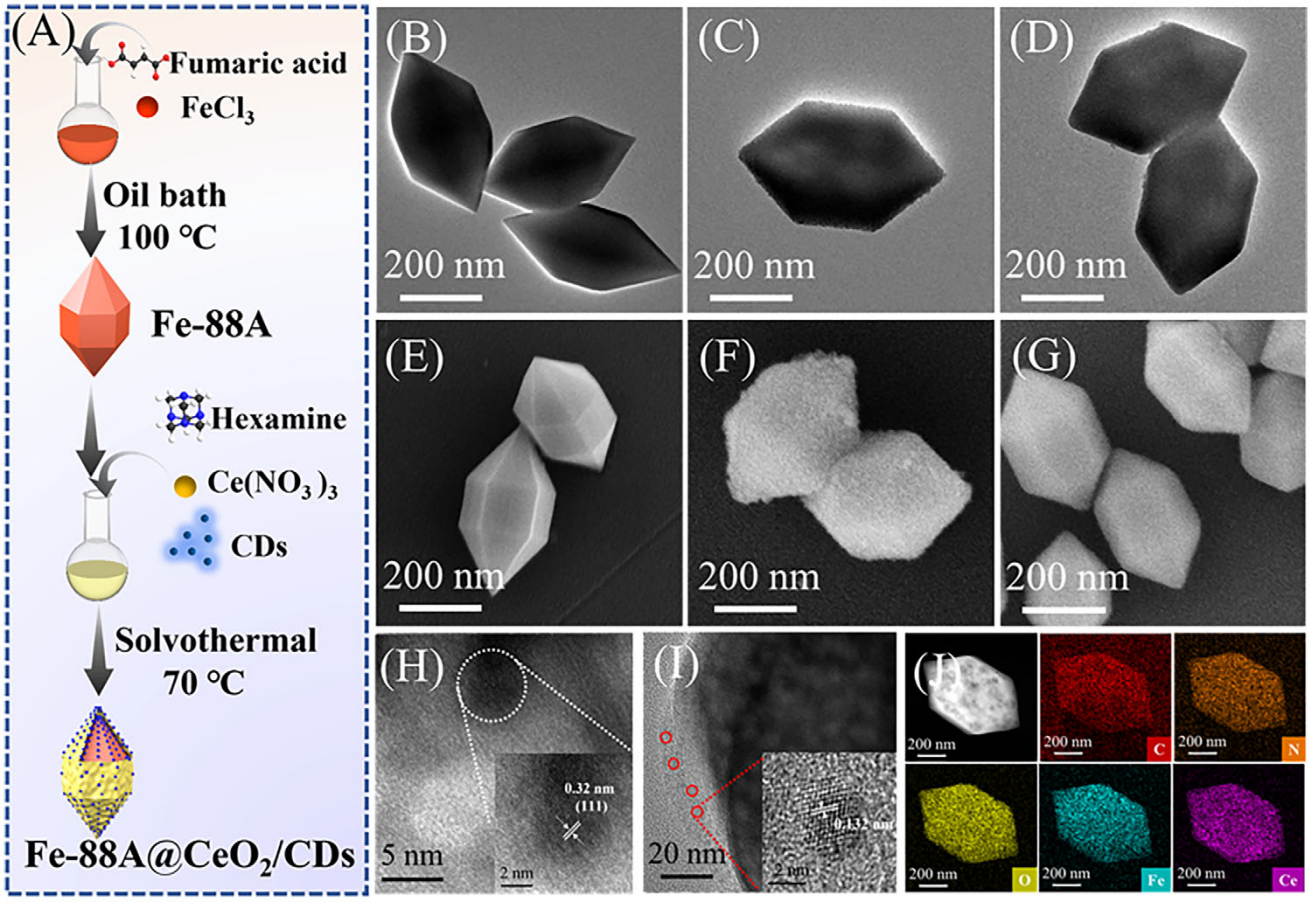
A) Schematic illustration of the synthesis process of Fe‐88A@CeO_2_/CDs; TEM B) and SEM E) of Fe‐88A; TEM C) and SEM F) of Fe‐88A@CeO_2_; TEM D) and SEM G) of Fe‐88A@CeO_2_/CDs; High‐resolution TEM images of Fe‐88A@CeO_2_/CDs: H) Lattice diagram of CeO_2_, I) Lattice diagram of CDs; J) High Angle Annular Dark Field image and Elemental mapping images of Fe, Ce, C, N and O of Fe‐88A@CeO_2_/CDs.

The synthesis route of Fe‐88A@CeO_2_/CDs nanozyme is bifurcated into two stages (**Figure** **1**A). First, homogeneous Fe‐88A was fabricated through a liquid growth procedure, transmission electron microscopy (TEM, Figure 1B) and scanning electron microscopy (SEM, Figure 1E) images demonstrate that the original Fe‐88A is uniform spindle‐shaped structure with a smooth surface. Subsequently, ammonium ions $\left({\mathrm{NH}}_{4}^{+}\right)$ from the C_6_H_12_N_4_ solution permeated the interior of Fe‐88A medium and reacted with Fe^3+^ ions within Fe‐88A. Fe‐88A was etched from the inside to the outside through a solvothermal process, which is conducive to incorporating nanoparticles into Fe‐88A. Meanwhile, CeO_2_ nanoparticles were gradually formed and loaded onto the spindle‐shaped Fe‐88A by Equations (1) and (2).^[^ ^46^ ^]^

(1) $$C_{6}H_{12}N_{4}+10H_{2}{O=6\mathrm{HCHO}+4\mathrm{NH}}_{4}^{+}+4OH^{-}$$

(2) $$2Ce^{3+}+6OH^{-}+O_{2}=2\mathrm{Ce}O_{2}+3H_{2}O$$

TEM (Figure 1C) and SEM (Figure 1F) images indicate that the prepared Fe‐88A@CeO_2_ nanozymes still maintain a spindle‐shaped morphology, and the surface gradually turns rough. The surface of Fe‐88A became rougher (Figure S1, Supporting Information) with the increase in the quantity of Ce(NO_3_)_3_·6H_2_O, testifying that CeO_2_ nanoparticles were coated onto Fe‐88A. Figure S2 (Supporting Information) reveals that the fabricated CDs exhibit excellent dispersion and a uniform size of ≈3 nm. During the growth of CeO_2_, CDs were also loaded into Fe‐88A. After introducing CDs into Fe‐88A, it was clearly observed from the TEM (Figure 1D) and SEM (Figure 1G) images that the surface of Fe‐88A@CeO_2_/CDs is smoother than that of Fe‐88A@CeO_2_. With the increase in the quantity of CDs, the surface of Fe‐88A@CeO_2_/CDs became even smoother, thereby confirming the successful loading of CDs into Fe‐88A (Figure S3, Supporting Information). In contrast, Fe‐88A@CeO_2_ was first synthesized and followed by the adsorption of CDs on the surface of Fe‐88A@CeO_2_. It was observed that the resultant Fe‐88A@CeO_2_@CDs (adsorption) remains a rough surface with aggregated CDs particles (Figure S4A,B, Supporting Information). High‐resolution transmission electron microscope (HRTEM) images (Figure 1H) were used to further validate the successful incorporation of CeO_2_ and CDs into Fe‐88A. The lattice spacing of 0.32 nm corresponds to the (111) plane of CeO_2_.^[^ ^46^ ^]^ In addition, Figure 1I exhibits the lattice spacing of 0.13 nm corresponding to the lattice spacing on the graphite (002) plane,^[^ ^47^ ^]^ suggesting that CDs have been successfully loaded into Fe‐88A. The element mapping images (Figure 1J) clearly exhibit the uniform distribution of Fe, Ce, C, N and O of the spindle‐shaped Fe‐88A@CeO_2_/CDs. As clearly shown in Figure S5A,B (Supporting Information), there is a significant difference in the distribution of carbon and cerium between Fe‐88A@CeO_2_/CDs (where carbon dots are incorporated via in situ growth) and Fe‐88A@CeO_2_@CDs (adsorption) (where carbon dots are introduced through adsorption). This observation further supports the in situ incorporation of carbon dots.

**Figure 2:**
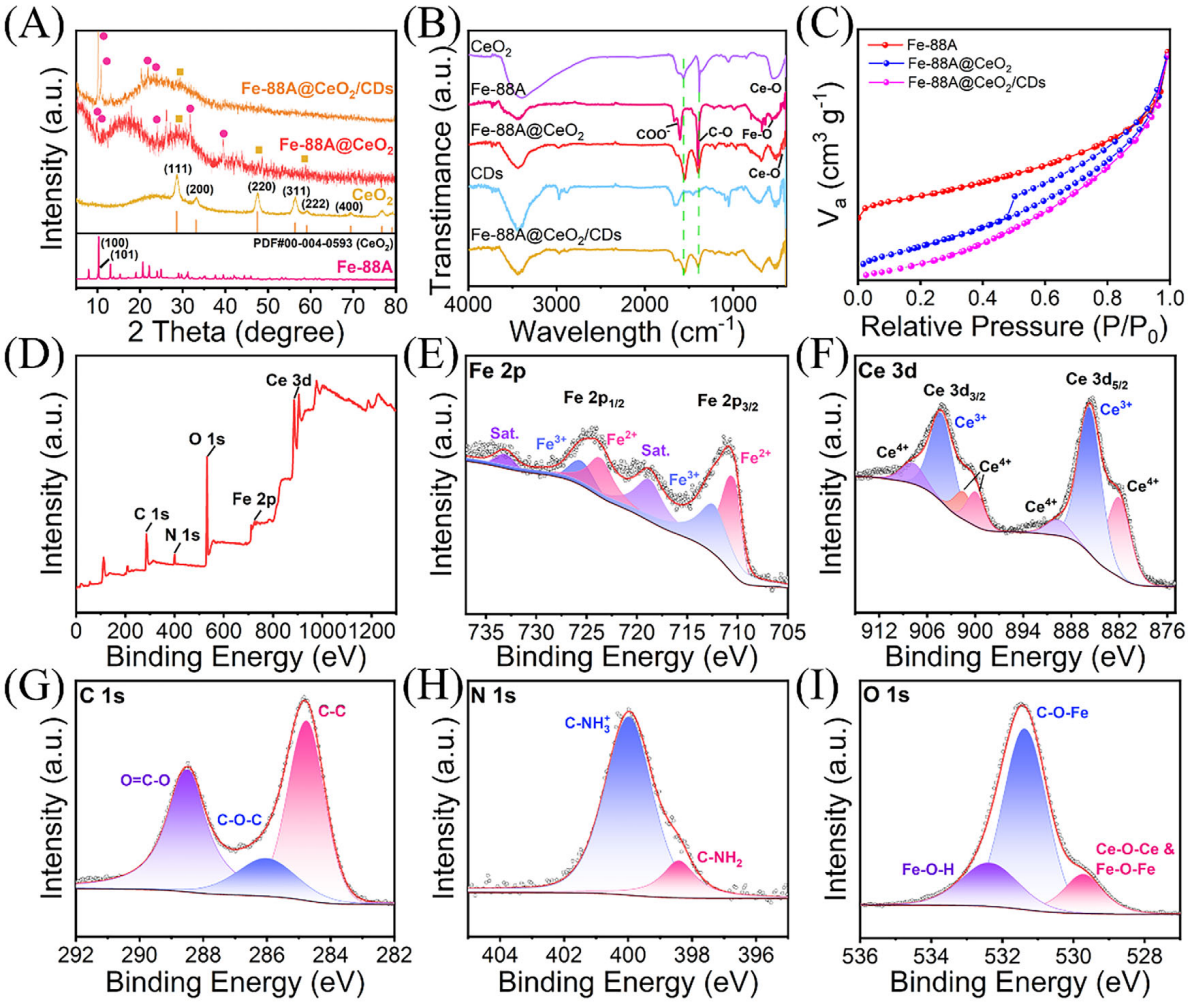
A) XRD patterns of Fe‐88A, CeO_2_, Fe‐88A@CeO_2_ and Fe‐88A@CeO_2_/CDs; B) FTIR spectra of CeO_2_, Fe‐88A, Fe‐88A@CeO_2_, CDs and Fe‐88A@CeO_2_/CDs; C) N_2_ adsorption/desorption isotherms of Fe‐88A, Fe‐88A@CeO_2_ and Fe‐88A@CeO_2_/CDs; D) Full XPS spectrum and high‐resolution XPS spectra of E) Fe 2p, F) Ce 3d, G) C 1s, H) N 1s, I) O 1s for Fe‐88A@CeO_2_/CDs.

**Figure** **2**A presents the X‐ray diffraction (XRD) spectrum of the synthesized nanozymes. The diffraction peaks at 10.2° and 11.5° of the original Fe‐88A correspond to the (100) and (101) crystal planes, respectively. The diffraction peaks at 28.6°, 33.1°, 47.5°, 56.3°, and 59.1° are attributed to the (111), (200), (220), (311), and (222) crystal faces of CeO_2_ (JCPDS 004‐0593). The coexistence of the diffraction peaks of Fe‐88A and CeO_2_ indicates the formation of Fe‐88A@CeO_2_. Interestingly, compared with Fe‐88A, the main diffraction peaks related to Fe‐88A in Fe‐88A@CeO_2_ shift due to the presence of CeO_2_. In the XRD pattern of Fe‐88A@CeO_2_/CDs, a broad diffraction peak was observed between 20° and 40°, besides the diffraction peaks of Fe‐88A and CeO_2_. This broad peak can be attributed to the (002) plane of the graphite lattice of CDs (Figure S6, Supporting Information).^[^ ^47^ ^]^ This result is consistent with the HRTEM and further confirms the successful loading of CDs.

Fourier transform infrared spectroscopy (FTIR) is presented in Figure 2B. The principal absorption bands of Fe‐88A were situated at ≈670 cm^−1^ (Fe─O), 1673 cm^−1^ (O─C─O), and 1429 cm^−1^ (C─O).^[^ ^48^ ^]^ After the integration of CeO_2_, the resulting Fe‐88A@CeO_2_ displays a prominent Ce─O bond peak at ≈520 cm^−1^,^[^ ^49^ ^]^ indicating that CeO_2_ has been successfully loaded. As anticipated, the FTIR spectrum of the prepared CDs displays the presence of nitrogen–oxygen functional groups. After loading CDs, Fe‐88A@CeO_2_/CDs presents similar peaks at 1659, 1545, and 1390 cm^−1^. Compared with Fe‐88A, its O─C═O vibration peak shifted from 1699 to 1673 cm^−1^. The C─O vibration peak shifted from 1429 to 1360 cm^−1^. This is possibly due to the electrical interaction among Fe‐88A, CeO_2_, and CDs. In addition, an analysis of Fe‐88A@CeO_2_/CDs was conducted by Zeta potential. In Figure S7A (Supporting Information), it is noted that the Fe‐88A@CeO_2_ exhibits significantly higher negative Zeta potential than that of Fe‐88A (‐11.0 mV), and the value is concentration‐dependent with the increase of Ce(NO_3_)_3_·6H_2_O. Similarly, as depicted in Figure S7B (Supporting Information), the Zeta potential of CDs is ‐10.2 mV, while the Zeta potential of Fe‐88A@CeO_2_ is evidently more negative. Interestingly, the Zeta potential of Fe‐88A@CeO_2_/CDs lies within the range of CDs and Fe‐88A@CeO_2_. This indicates that the formation of Fe‐88A@CeO_2_/CDs affected the surface charge of Fe‐88A@CeO_2_, which provides further evidence for the successful preparation of Fe‐88A@CeO_2_/CDs. The more negative Zeta potential (compared to pure Fe‐88A) facilitates binding the positively charged substrate TMB.

The specific surface area (Figure 2C) and average pore size (Figure S8, Supporting Information) of Fe‐88A, Fe‐88A@CeO_2_, and Fe‐88A@CeO_2_/CDs were evaluated using N_2_ adsorption–desorption measurements. The results indicate that the synthesized nanozymes exhibit a type IV isotherm, suggesting the presence of mesoporous structures. The specific surface areas of Fe‐88A, Fe‐88A@CeO_2_, and Fe‐88A@CeO_2_/CDs are 42.04, 99.96, and 64.56 m^2^ g^−1^, respectively. The corresponding average pore sizes are 5.03, 9.97, and 14.37 nm, respectively (Table S1, Supporting Information). These changes in specific surface area and pore size for Fe‐88A@CeO_2_ can be attributed to the etching effect of hexamethylenetetramine (HMTA) during the loading of CeO_2_. The decrease in specific surface area of Fe‐88A@CeO_2_/CDs is likely due to the infiltration of CDs into the porous structure of Fe‐88A. Larger pore size is advantageous for the diffusion of nanozyme substrate and the adsorption of more dissolved oxygen, thereby enhancing the oxidase (OXD)‐like activity of nanozymes.^[^ ^50^ ^]^

The elemental composition and chemical state of Fe‐88A@CeO_2_@CDs were characterized through X‐ray photoelectron spectroscopy (XPS). Figure S9A (Supporting Information) illustrates the presence of C, O, and N elements in XPS of CDs. The high‐resolution XPS spectra of each element further confirm that CDs are rich in nitrogen–oxygen functional groups (Figure S9B–D). In the full XPS spectrum of the Fe‐88A@CeO_2_/CDs (Figure 2D), it can be observed that there are peaks of Fe, Ce, C, N, and O elements, which is in accordance with the result of the EDS mapping (Figure S10, Supporting Information). In the high‐resolution XPS spectrum of Fe 2p for Fe‐88A@CeO_2_/CDs (Figure 2E), it is evident that iron exists in the chemical states of Fe^3+^ (711.7 and 725.2 eV) and Fe^2+^ (710.5 and 723.8 eV).^[^ ^49^ ^]^ The high‐resolution spectrum of Ce 3d in XPS (Figure 2F) reveals that Ce^3+^ (903.1 and 884.4 eV) and Ce^4+^ (881.6, 889.7, 899.1, 901.5, and 909.8 eV)^[^ ^51^ ^]^ coexist in Fe‐88A@CeO_2_/CDs. The coexistence of Fe (II)/Fe (III) and Ce (III)/Ce (IV) redox couples in Fe‐88A@CeO_2_/CDs nanozyme constitutes a dual electron transfer channel to enhance the electron transport efficiency. The high‐resolution XPS spectrum of the C1s (Figure 2G) can be decomposed into three separate peaks at 284.7, 286.3, and 288.8 eV, corresponding to the C─C, C─O─C, and O═C─O bonds respectively. The high‐resolution XPS spectrum of N 1s (Figure 2H) indicates the presence of an amino group at 399.2 eV (C‐NH_2_) and 400.6 eV (C‐NH^3+^), which is attributed to the growth of CeO_2_ and the abundance of nitrogen‐containing groups on CDs.^[^ ^50^ ^]^ The high‐resolution XPS spectrum of O 1s (Figure 2I) reveals the presence of three types of oxygen.^[^ ^49^ ^]^ The fitting peaks at 529.3, 531.4 and 532.5 eV are ascribed to Fe─O─Fe/Ce─O─Ce, C─O─Fe, and Fe─O─H bonds, respectively. It is worth noting that the C─O─Fe bond indicates the presence of adsorbed oxygen or defective oxygen.

### 2.2 Oxidase‐Like Activity and Steady‐State Kinetics of Fe‐88A@CeO_2_/CDs Nanozyme

**Figure 3:**
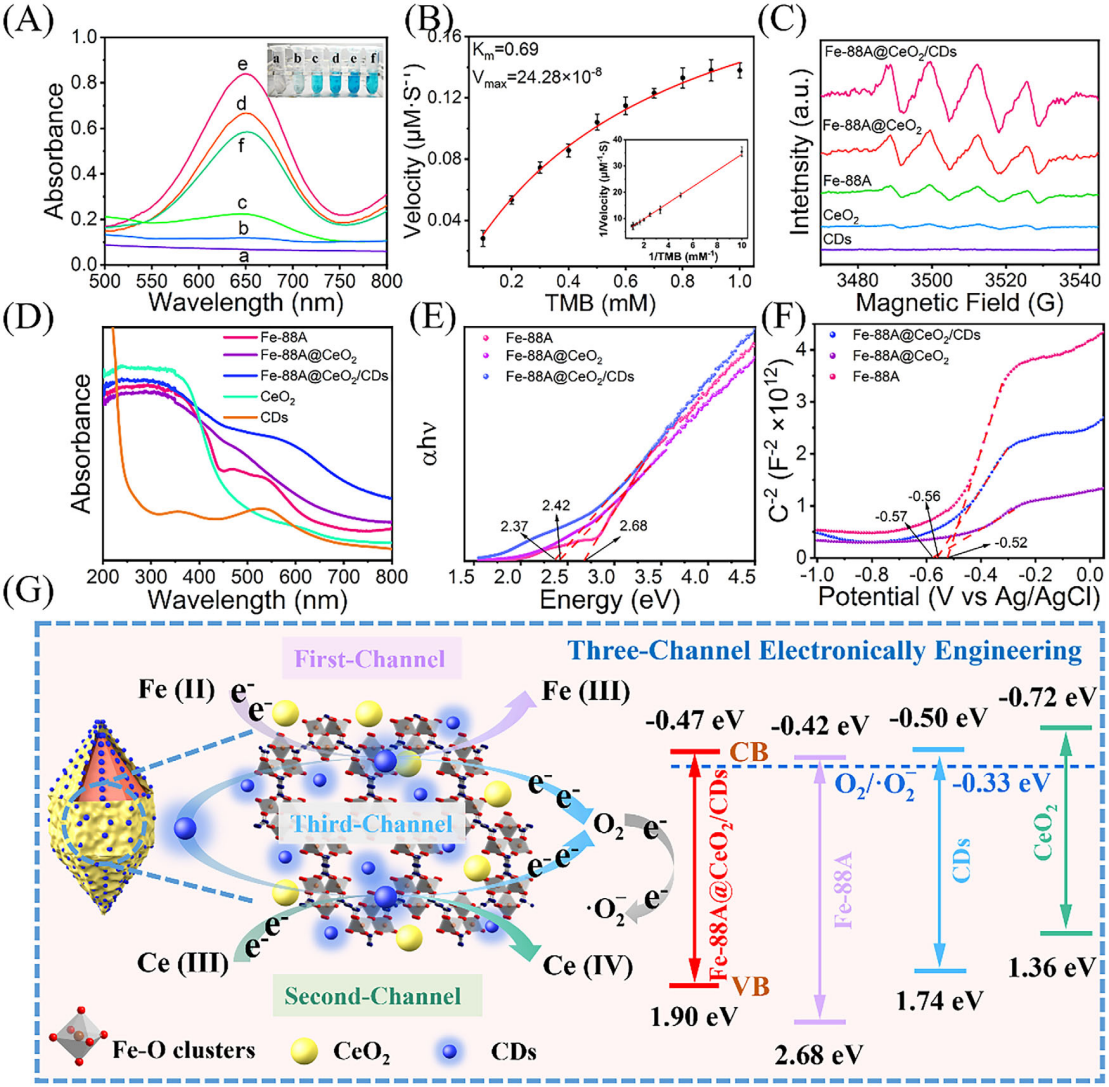
A) Oxidase‐like activity of the prepared nanozymes (a: CDs+TMB, b: CeO_2_+TMB, c: Fe‐88A+TMB, d: Fe‐88A@CeO_2_+TMB, e: Fe‐88A@CeO_2_/CDs+TMB, f: Fe‐88A@CeO_2_@CDs (adsorption)+TMB; B) The kinetics curves of Fe‐88A@CeO_2_/CDs with TMB as substrate, and the inset shows the corresponding double reciprocal plots of Fe‐88A@CeO_2_/CDs; C) EPR spectra of CDs, CeO_2_, Fe‐88A, Fe‐88A@CeO_2_, Fe‐88A@CeO_2_/CDs; D) UV–vis diffuse reflectance spectra of CDs, CeO_2_, Fe‐88A, Fe‐88A@CeO_2_ and Fe‐88A@CeO_2_/CDs and corresponding Tauc plots E). F) Mott–Schottky plots and the potential is versus Ag/AgCl for Fe‐88A, Fe‐88A@CeO_2_, and Fe‐88A@CeO_2_/CDs. G) The mechanistic process of the conversion of O_2_ to ·$O_{2}^{-}$ catalyzed by three‐channel electron transfer engineered Fe‐88A@CeO_2_/CDs nanozyme.

Utilizing TMB as the substrate, the OXD‐like activity of the nanozymes was investigated. As depicted in **Figure** **3**A, there is no absorption when CDs were mixed with TMB (curve a), indicating that CDs cannot oxidize TMB in the absence of an external stimulus. Moreover, only weak absorption peaks occurred at 652 nm accompanied with light blue TMB solution when TMB was mixed with CeO_2_ or Fe‐88A (curves b,c), suggesting the weak OXD‐like activity of single CeO_2_ or Fe‐88A. It is notable that a strong absorption at 652 nm and dark blue were observed in the reaction solutions of TMB and Fe‐88A@CeO_2_ (curve d), suggesting the excellent OXD‐like activity. Intriguingly, the solution of TMB and Fe‐88A@CeO_2_@CDs shows the strongest absorption (curve e), and the color of the solution becomes darker, demonstrating greatly enhanced OXD‐like activity of Fe‐88A@CeO_2_@CDs. However, the absorbance of TMB and Fe‐88A@CeO_2_@CDs (adsorption) is lower than that of Fe‐88A@CeO_2_. This phenomenon is attributed to the accumulation of a substantial number of CDs on the surface of Fe‐88A@CeO_2_, leading to the inhibition of iron and cerium catalytic sites. The results further demonstrate that introducing CDs into Fe‐88A during the growth process of CeO_2_ is beneficial for enhancing OXD‐like activity of Fe‐88A@CeO_2_. Moreover, the amount of Ce(NO_3_)_3_·6H_2_O and CDs were optimized and Fe‐88A@CeO_2_/CDs demonstrates the optimum catalytic activity at the Ce(NO_3_)_3_·6H_2_O quality of 0.15 g and the CDs quality of 10 mg (Figure S11A,B, Supporting Information). The catalytic capacity of nanozymes is also influenced by the reaction environment. Therefore, the temperature of the enzymatic reaction was optimized (Figure S11C, Supporting Information), and the temperature of 30 °C is chosen as the optimal condition for Fe‐88A@CeO_2_/CDs nanozyme catalysis.

The steady‐state kinetics of Fe‐88A@CeO_2_/CDs was examined by varying different concentrations of TMB. It was discovered that Fe‐88A@CeO_2_/CDs nanozyme followed the classical Michaelis–Menten model and Lineweaver–Burk model. The calculated *K*_m_ and *V*_max_ values of Fe‐88A@CeO_2_/CDs nanozyme for TMB (Figure 3B) are 0.69 mm and 24.28 × 10^−8^ m·s^−1^, respectively, indicating that Fe‐88A@CeO_2_/CDs nanozyme has a strong affinity and catalytic rate for TMB. In contrast, the *K*_m_ and *V*_max_ of Fe‐88A@CeO_2_ nanozyme for TMB (Figure S12A, Supporting Information) are 0.74 mm and 11.12 × 10^−8^ m·s^−1^, respectively. These results imply that Fe‐88A@CeO_2_/CDs nanozyme has higher affinity and catalytic rate for TMB. Fe‐88A@CeO_2_/CDs nanozyme exhibits superior performance compared to other nanozymes (Table S2, Supporting Information). The results demonstrate that Fe‐88A@CeO_2_/CDs nanozyme possesses excellent OXD‐like activity for broad application prospects in the construction of biosensors.

### 2.3 Catalytic Mechanisms of Fe‐88A@CeO_2_/CDs Nanozyme

The catalytic mechanism of Fe‐88A@CeO_2_/CDs nanozyme was investigated. It can be observed that the catalytic activity of Fe‐88A@CeO_2_/CDs was inhibited in the absence of O_2_ (Figure S12B, Supporting Information). Based on previous studies, nanozymes with OXD‐like activity typically convert O_2_ into reactive oxygen species and utilize them to oxidize substrates such as TMB.^[^ ^52^, ^53^ ^]^ In general, O_2_ was initially adsorbed on the surface of Fe‐88A@CeO_2_/CDs nanozyme, and then O_2_ was reduced to ${\mathrm{HO}}_{2}^{\cdot }$ by the Fe and Ce sites of nanozyme (Equations 3 and 4). Subsequently, ${\mathrm{HO}}_{2}^{\cdot }$ decomposed to generate ·$O_{2}^{-}$ (Equation 5), which further oxidized TMB to form a blue product (Equation 6). The possible catalytic mechanism of Fe‐88A@CeO_2_/CDs for TMB is as follows:

(3) $$Fe^{2+}+O_{2}+H_{3}O^{+}={\mathrm{HO}}_{2}^{\cdot }+Fe^{3+}+H_{2}O$$

(4) $$Ce^{3+}+O_{2}+H_{3}O^{+}={\mathrm{HO}}_{2}^{\cdot }+Ce^{4+}+H_{2}O$$

(5) $${\mathrm{HO}}_{2}^{\cdot }=\cdot O_{2}^{-}+H^{+}$$

(6) $$\mathrm{TMB}+\cdot O_{2}^{-}=\mathrm{Blue}\;\mathrm{product}\;(\mathrm{ox}-\mathrm{TMB})$$

We selected p‐benzoquinone (PBQ) as a radical scavenging agent to confirm the generation of superoxide anion radicals (·$O_{2}^{-}$). As illustrated in Figure S13A,B (Supporting Information), the reaction activity decreased significantly following the addition of PBQ, indicating that ·$O_{2}^{-}$ is the predominant reactive oxygen species produced during the reaction. Notably, in the case of the Fe‐88A@CeO_2_/CDs system, the effect of PBQ on catalytic activity was particularly pronounced, which indirectly suggests an enhancement in enzymatic‐like catalytic performance. Additionally, ·$O_{2}^{-}$ was identified by electron paramagnetic resonance (EPR) using DMPO as a spin probe. As can be observed from Figure 3C, no obvious signals of ·$O_{2}^{-}$ were observed for CDs, while single Fe‐88A or CeO_2_ show weak signals related to ·$O_{2}^{-}$, indicating the poor OXD‐like activity of Fe‐88A or CeO_2_. After loading CeO_2_ onto Fe‐88A, Fe‐88A@CeO_2_ exhibits strong ·$O_{2}^{-}$ signals compared with CeO_2_ and Fe‐88A, indicating the enhanced OXD‐like activity of Fe‐88A@CeO_2_. This is because more active sites and electron channels of Fe‐88A@CeO_2_ accelerate electron transfer from O_2_ to ·$O_{2}^{-}$. Furthermore, much stronger signals were observed from Fe‐88A@CeO_2_/CDs, suggesting that the loading of CDs into Fe‐88A@CeO_2_ provides a further enhanced OXD‐like activity. The outstanding electron transport ability of CDs facilitates the electron transfer process between the reaction of O_2_ and ·$O_{2}^{-}$. These factors all contribute to the rapid oxidation of TMB by ·$O_{2}^{-}$.

The three‐channel electron transfer process was examined in detail through UV–vis diffuse reflectance spectrum and electrochemical studies. As shown in Figure 3D, Fe‐88A, Fe‐88A@CeO_2_, and Fe‐88A@CeO_2_/CDs exhibit maximum absorption peaks at ≈350 nm, which may be attributed to charge‐transfer from the ligand O (II) to the metal Fe (III).^[^ ^47^ ^]^ The corresponding Tauc plot is derived from Equation 7:

(7) $$\alpha \mathrm{hv}=A{\left(\mathrm{HV}-\mathrm{Eg}\right)}^{n/2}$$

Thus, the bandgap energies (*E*_g_) of CeO_2_, CDs, Fe‐88A, Fe‐88A@CeO_2_, and Fe‐88A@CeO_2_/CDs are calculated to be 2.08, 2.24, 2.68, 2.42, and 2.37 eV, respectively (Figure 3E; Figure S14A, Supporting Information). These results indicate that the incorporation of CeO_2_ and CDs into Fe‐88A leads to a gradual redshift of the absorption edge and a reduction in bandgap energy, which facilitates electron transfer and enhances the generation of ·$O_{2}^{-}$. According to Mott–Schottky analysis, the flat‐band potentials (*E*_fb_) of CeO_2_, CDs, Fe‐88A, Fe‐88A@CeO_2_, and Fe‐88A@CeO_2_/CDs are ‐0.82, ‐0.60, ‐0.52, ‐0.56 and ‐0.57 V, respectively (Figure 3F; Figure S14B, Supporting Information).Therefore, the calculated *E*_CB_ values of CeO_2_, CDs, Fe‐88A, Fe88A@CeO_2_, and Fe‐88A@CeO_2_/CDs are ‐0.72, ‐0.50, ‐0.42, ‐0.46, and ‐0.47 V, respectively.^[^ ^54^ ^]^ As clearly observed in Figure S15 (Supporting Information), Fe‐88A@CeO_2_/CDs exhibits a more negative conduction band potential compared to Fe‐88A and Fe‐88A@CeO_2_, which indicates a higher electron donor density. This observation highlights the synergistic role of CeO_2_ and carbon dots (CDs) in modulating the electronic structure of the nanozyme. Consequently, it can be inferred that Fe‐88A@CeO_2_/CDs is capable of producing a greater amount of ·$O_{2}^{-}$, a conclusion that aligns well with both the EPR spectroscopic results and the comparative analysis of enzyme‐like activity.^[^ ^55^, ^56^ ^]^ Moreover, electrochemical impedance spectroscopy (EIS) was employed to evaluate the electron transfer rate (Figure S16A,B, Supporting Information). The decreased semicircle diameter observed for Fe‐88A@CeO_2_/CDs indicates faster charge‐transfer.

Collectively, Fe‐88A@CeO_2_/CDs demonstrate superior OXD‐like activity due to the following mechanism (illustrated in Figure 3G): The synergistic catalysis between Fe (II)/Fe (III) and Ce (III)/Ce (IV) forms an efficient double electron transfer channel, while CDs produce another electron transfer channel. Three‐channel electron transfer engineering markedly reduces the bandgap energy of Fe‐88A@CeO_2_/CDs and decreases the energy barrier for electron migration. The introduction of three‐channel electron transfer engineering facilitates the participation of a larger number of electrons in the reduction of O_2_, thereby significantly enhancing the OXD‐like activity of Fe‐88A@CeO_2_/CDs.

### 2.4 Fluorescence Properties and Quenching Mechanism of Fe‐88A@CeO_2_/CDs

**Figure 4:**
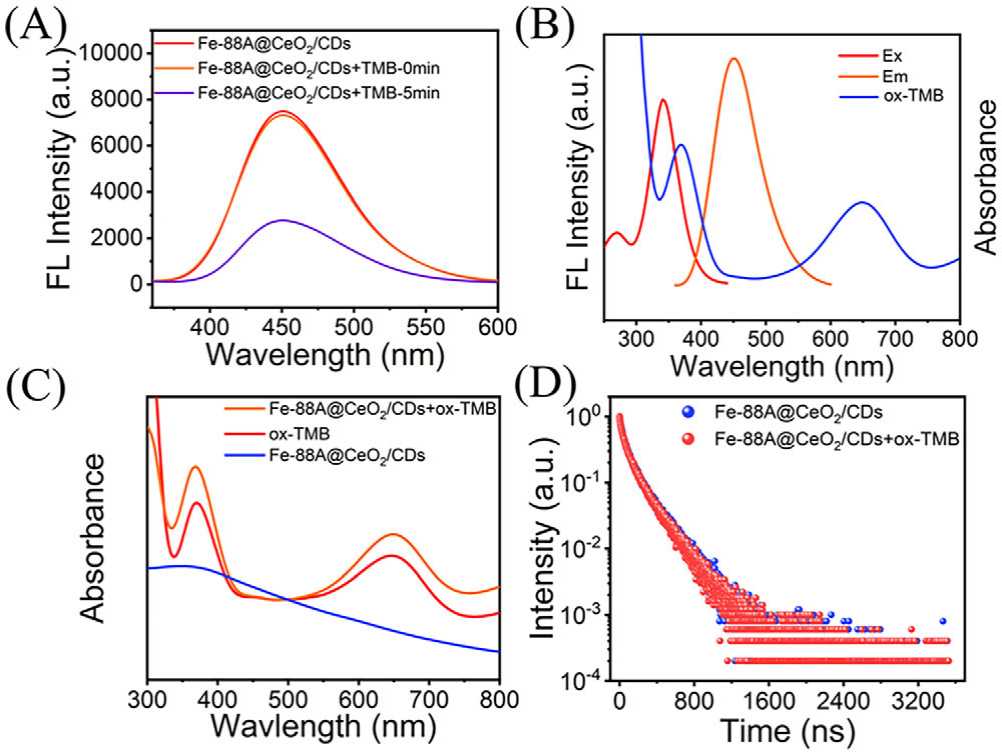
A) Fluorescence spectra of Fe‐88A@CeO_2_/CDs, Fe‐88A@CeO_2_/CDs+TMB (0 min), and Fe‐88A@CeO_2_/CDs+TMB (5 min); B) Fluorescence excitation and emission spectra of Fe‐88A@CeO_2_/CDs and UV–vis absorption spectrum of ox‐TMB; C) UV–vis absorption spectra of ox‐TMB, Fe‐88A@CeO_2_/CDs+ox‐TMB and Fe‐88A@CeO_2_/CDs; (D) Fluorescence lifetimes of Fe‐88A@CeO_2_/CDs and Fe‐88A@CeO_2_/CDs+ox‐TMB.

The fluorescence properties of Fe‐88A@CeO_2_/CDs were investigated by fluorescence emission spectroscopy. As shown in Figure S17 (Supporting Information), Fe‐88A@CeO_2_/CDs displays excitation dependence, and the optimal excitation and emission wavelengths are 340 and 452 nm, respectively. It was notable that the inherent fluorescence of Fe‐88A@CeO_2_/CDs was not influenced by TMB itself, and the fluorescence was significantly quenched after co‐incubation (**Figure** **4**A), indicating that ox‐TMB leads to the fluorescence quenching. To determine the quenching mechanism, it can be observed that there is a considerable overlap between the excitation spectrum of Fe‐88A@CeO_2_/CDs and the absorption spectrum of ox‐TMB (Figure 4B), which is a prerequisite for the formation of the internal filtration effect (IFE) between Fe‐88A@CeO_2_/CDs and ox‐TMB. No new UV–vis absorption peak emerged after the reaction, indicating that no new complex formed during the quenching process (Figure 4C), which excludes the possibility of static quenching. The fluorescence lifetime of Fe‐88A@CeO_2_/CDs exhibits minimal change upon the addition of ox‐TMB. The slight alteration in fluorescence lifetime suggests that no significant energy resonance transfer occurred between Fe‐88A@CeO_2_/CDs and ox‐TMB. (Figure 4D). These results confirmed that the quenching mechanism is caused by the IFE.

### 2.5 Characterization of Nanozyme Probe and Feasibility of Colorimetric‐Fluorescence Bimodal Detection of SEB

**Figure 5:**
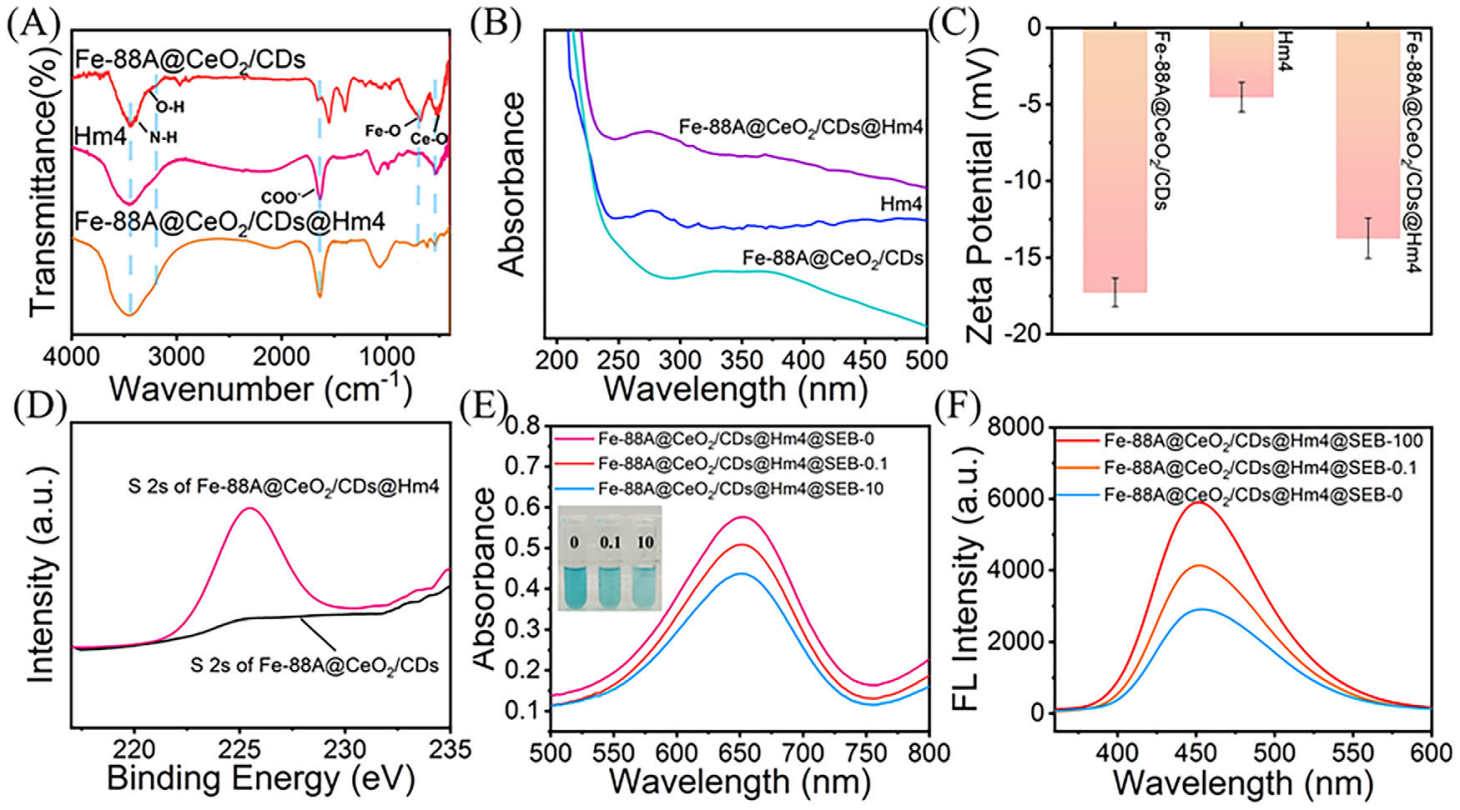
A) FTIR spectra, B) UV–vis absorption spectra and C) Zeta potential of Fe‐88A@CeO_2_/CDs, Hm4 and Fe‐88A@CeO_2_/CDs@Hm4; D) XPS spectra of Fe‐88A@CeO_2_/CDs and Fe‐88A@CeO_2_/CDs@Hm4 probe; E,F) Feasibility of this proposed SEB assay based on the UV–vis absorption and fluorescence spectra, respectively.

A label‐free colorimetric‐fluorescence bimodal immunosensor was fabricated by employing Fe‐88A@CeO_2_/CDs@Hm4 as a nanozyme probe. **Figure** **5**A displays the FTIR spectrum of the Fe‐88A@CeO_2_/CDs@Hm4. The stretching vibration of COO^−^ at 1625 cm^−1^ in the Hm4 antibody was also observed in FTIR spectrum of Fe‐88A@CeO_2_/CDs@Hm4. Additionally, the stretching vibrations of Fe─O and Ce─O are still detectable in Fe‐88A@CeO_2_/CDs@Hm4, indicating that the Hm4 antibody was successfully modified on Fe‐88A@CeO_2_/CDs. Figure 5B presents the UV–vis absorption of the nanozyme probe. The UV absorption curve of the Hm4 antibody possesses a notable absorption peak at 280 nm. Compared to the Fe‐88A@CeO_2_/CDs nanozyme, the Fe‐88A@CeO_2_/CDs@Hm4 nanozyme probe displays the characteristic absorption peak of the proteins similar to the Hm4 antibody. The shift of the absorption peak is attributed to the interaction between Fe‐88A@CeO_2_/CDs and the Hm4 antibody. The immobilization of Hm4 on nanozyme was verified through Zeta potential (Figure 5C). The potentials of Fe‐88A@CeO_2_/CDs, Hm4, and Fe‐88A@CeO_2_/CDs@Hm4 are ‐17.2, ‐4.5, and ‐13.7 mV, respectively. The corresponding change in Zeta potential is attributed to the successful assembly of Hm4. The XPS peaks of the nanozyme probe and Fe‐88A@CeO_2_/CDs were presented in Figure 5D. There is no S 2s peak in the XPS spectrum of Fe‐88A@CeO_2_/CDs nanozyme, while a S 2s peak in the nanozyme probe was ascribed to the existence of sulfur in the antibody biomolecules. All the aforementioned results indicate the successful preparation of Fe‐88A@CeO_2_/CDs@Hm4 nanozyme probe.

The feasibility of label‐free colorimetric‐fluorescence bimodal detection of SEB based on Fe‐88A@CeO_2_/CDs@Hm4 nanozyme probe was investigated in Figure 5E,F. The Fe‐88A@CeO_2_/CDs@Hm4 nanozyme probe+TMB shows an intensive UV–vis absorption peak. After incubating various concentrations of SEB, the absorbance of ox‐TMB decreased gradually (Figure 5E). Owing to the specific recognition of SEB, the formed Hm4‐SEB immunocomplex inhibits the OXD‐like activity of Fe‐88A@CeO_2_/CDs, thereby reducing the formation of ox‐TMB. Concurrently, the generation of ox‐TMB effectively quenches the fluorescence of Fe‐88A@CeO_2_/CDs (Figure 5F). As the immunocomplex increases, the generation of ox‐TMB decreases, thereby weakening the quenching effect and restoring the fluorescence. The optimal incubation time for binding SEB was 20 min (Figure S18, Supporting Information). The feasibility study indicates that the Fe‐88A@CeO_2_/CDs@Hm4 probe can achieve label‐free colorimetric‐fluorescence bimodal immunoassay of SEB.

### 2.6 Performance of Colorimetric‐Fluorescence Bimodal Detection of SEB

**Figure 6:**
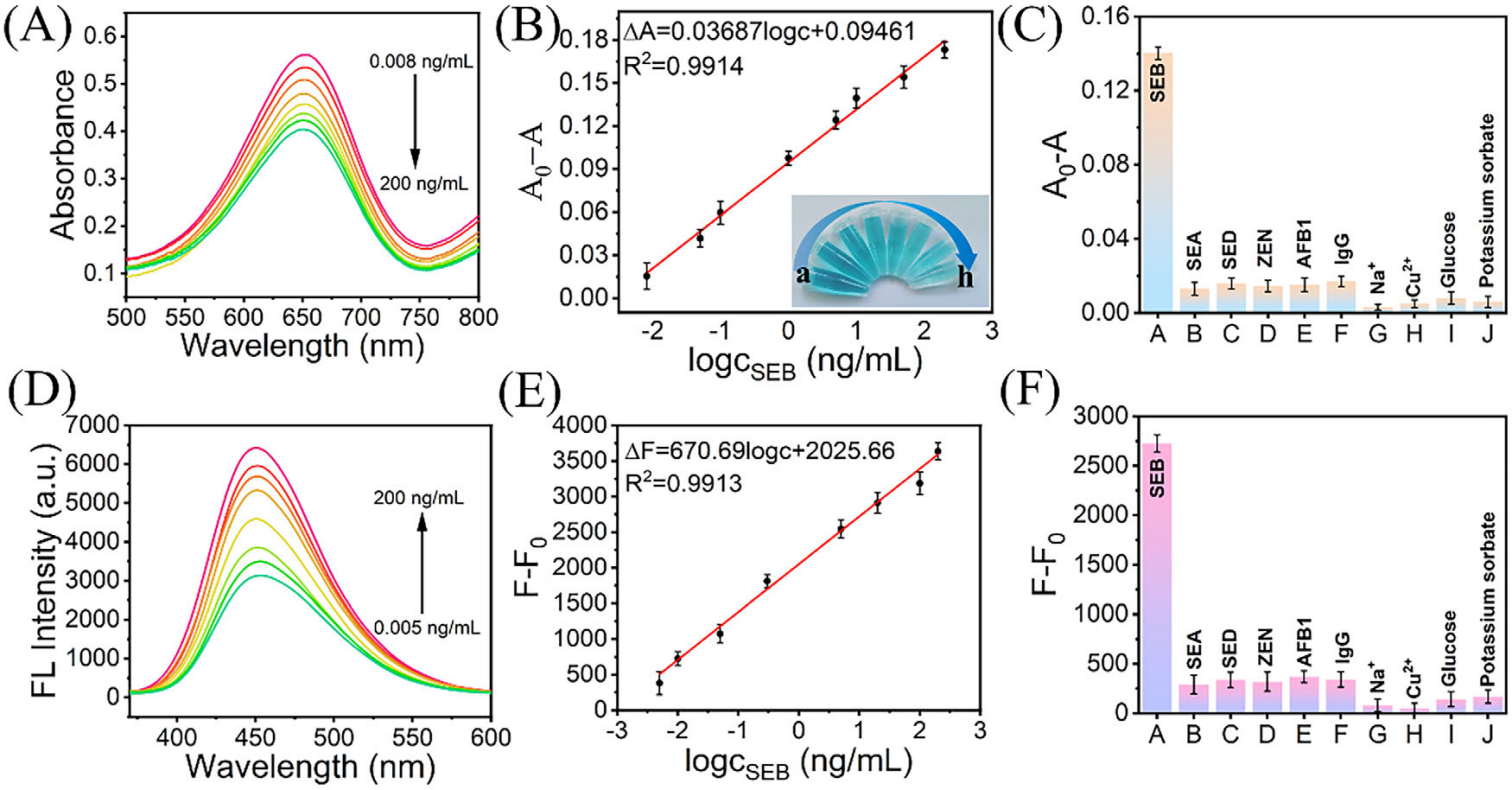
A) UV–vis absorption of Fe‐88A@CeO_2_/CDs@Hm4 with TMB in the presence of different concentrations of SEB, and B) calibration curve plotted by A_0_‐A versus the SEB concentrations (from a to h: 0.008, 0.05, 0.1, 1, 5, 10, 50 and 200 ng mL^−1^ SEB); D) Fluorescence of Fe‐88A@CeO_2_/CDs@Hm4 with TMB in the presence of different concentrations of SEB (from 0.005 to 200 ng mL^−1^), and E) calibration curve plotted by F‐F_0_ versus the SEB concentrations; C, F) Specificity of the colorimetric and fluorometric detection of SEB, respectively (*n* = 3).

Under optimal experimental conditions, the analytical performances of the Fe‐88A@CeO_2_/CDs‐based bimodal immunosensor for detection of SEB are studied. The Fe‐88A@CeO_2_/CDs@Hm4 probe was exposed to PBS (pH 7.4) to bind SEB. For colorimetric mode, as the concentration of SEB increased from 0.008 to 200 ng mL^−1^, the absorbance value at 652 nm continuously decreased due to the inhibition effect of SEB antigens (**Figure** **6**A). Consequently, a favorable linear relationship was established between the absorbance and the logarithmic function of SEB concentration, and the corresponding calibration curve was expressed by the regression equation ΔA = 0.03687 logc+0.09461 with a R^2^ of 0.9914 (Figure 6B) (ΔA = A_0_‐A, where A_0_ is the absorbance of ox‐TMB at 652 nm for Fe‐88A@CeO_2_/CDs@Hm4 system; A is the absorbance of ox‐TMB at 652 nm after reaction of different concentrations of toxins with Fe‐88A@CeO_2_/CDs@Hm4). The limit of detection (LOD) was calculated to be 0.003 ng mL^−1^ (S/N = 3). The colorimetric method also provides a visual blue gradient for the detection of SEB (inset of Figure 6B).

Similarly, quantitative measurements of SEB can be accomplished by fluorescence mode. As the ox‐TMB catalyzed by Fe‐88A@CeO_2_/CDs@Hm4 quenches its fluorescence through internal filtering, the fluorescence intensity gradually enhanced when the concentration of the SEB increased from 0.005 to 200 ng mL^−1^ (Figure 6D). The corresponding calibration curve was represented by the regression equation ΔF = 670.69 logc+2025.66, with a R^2^ of 0.9913 (Figure 6E) (ΔF = F‐F_0_, where F_0_ is the fluorescence intensity of ox‐TMB at 452 nm in Fe‐88A@CeO_2_/CDs@Hm4 system; F is the fluorescence intensity of ox‐TMB at 452 nm after the reaction of different toxins with Fe‐88A@CeO_2_/CDs@Hm4 system). The fluorescence mode can achieve a LOD (S/N = 3) of 0.001 ng mL^−1^ for SEB. Notably, the proposed label‐free colorimetric‐fluorescence bimodal immunosensor has better analytical performance compared to previously reported methods (Table S3, Supporting Information).

### 2.7 Selectivity, Reproducibility, Stability, and Practicability of Colorimetric‐Fluorescence Bimodal Immunosensor

The selectivity of an immunosensor is the key factor for high‐sensitivity detection of the target antigen. The selectivity of the immunosensor plays a critical role in enabling high‐sensitivity detection of the target antigen. To further evaluate the anti‐interference capability of the proposed bimodal immunosensor for SEB detection, several common potential interfering substances were selected, including Staphylococcal enterotoxin A (SEA), Staphylococcal enterotoxin D (SED), zearalenone (ZEN), aflatoxin B1 (AFB1), and immunoglobulin G (IgG), all at a concentration of 10 ng mL^−1^, as well as representative inorganic ions (Na^+^, Cu^2+^) and food additives (glucose, potassium sorbate). These substances were employed to comprehensively assess the specificity and robustness of the sensing system under potentially interfering conditions. For the colorimetric method (Figure 6C), the absorbance change (ΔA) of the Fe‐88A@CeO_2_/CDs@Hm4/TMB system is slight after exposure to the other interfering substance samples. In the presence of SEB, a significant value of ΔA was clearly observed. For the fluorescence method (Figure 6F), the fluorescence intensity of Fe‐88A@CeO_2_/CDs@Hm4 changed slightly after exposure to the other interfering substance samples. In the presence of SEB, it is evident that ΔF increased greatly. These results indicate that the proposed bimodal immunosensor exhibits excellent specificity for detection of SEB.

To evaluate the reproducibility of the bimodal immunosensor, SEB was measured concurrently using five distinct immunosensors (Figure S19A,C, Supporting Information), and the relative standard deviations of the five measurements by colorimetry and fluorescence are 0.62% and 0.86%, respectively, suggesting the good reproducibility. Additionally, the stability of the fabricated bimodal immunosensor was investigated by storing the Fe‐88A@CeO_2_/CDs@Hm4 probe at 4 °C and measuring its absorbance and fluorescence intensity every 5 days. As depicted in Figure S19B,D (Supporting Information), the absorbance and fluorescence intensities remain 94.68% and 96.92% of the initial values after 20 days, indicating that the bimodal immunosensor demonstrates acceptable stability.

In order to illustrate the practicability of the proposed bimodal immunosensor in monitoring real samples, standard SEB antigens were spiked in real samples (including water and milk) for recovery experiments. To avoid a certain extent of interference in the discrimination of color and fluorescence, the milk sample was diluted 100 times after pretreatment. Each sample was tested three times using the proposed bimodal immunosensor (*n* = 3). As shown in Table S4 (Supporting Information), the recovery rates of the label‐free colorimetric‐fluorescence bimodal immunosensor are 94.83%‐102.70% and 98.80%‐110.00%, respectively. The results indicate that the constructed bimodal immunosensor has considerable practical value in monitoring toxin contamination in various food or water samples.

## 3 Conclusion

In summary, a novel three‐channel electron engineering was proposed to construct a versatile Fe‐88A@CeO_2_/CDs nanozyme with enhanced oxidase‐like activity for efficient biomimetic catalysis. The Fe‐88A@CeO_2_/CDs nanozyme was prepared by incorporation of CeO_2_ and CDs into the porous structure of Fe‐88A, and characterized in detail using various means. After doping CeO_2_ into Fe‐88A, the synergistic catalysis between Fe (II)/Fe (III) and Ce (III)/Ce (IV) forms an effective double electron transfer channel. The doping of CDs endows the nanozyme with another electron transfer channel. The three‐channel engineering regulates the bandgap energy and restricted active sites of Fe‐88A@CeO_2_/CDs and further accelerates electron transfer process in catalytic reaction, thereby significantly boosting the OXD‐like activity of Fe‐88A@CeO_2_/CDs for biomimetic catalysis. The nanozyme can rapidly catalyze TMB substrate to form blue ox‐TMB in the presence of O_2_. ox‐TMB can quench the fluorescence of CDs in the Fe‐88A@CeO_2_/CDs through the internal filtering effect. As verification, Fe‐88A@CeO_2_/CDs nanozyme was utilized to develop an efficient label‐free colorimetric‐fluorescence bimodal immunosensor. Once bimodal immunosensor specifically bind the SEB, a decrease in the absorption of ox‐TMB and the recovery of the fluorescence signal were achieved for quantitative and specific detection of SEB. This research proposes a promising strategy to construct nanozyme with enhanced OXD‐like activity for effective biomimetic catalysis, providing new thought for developing powerful immunosensors, which can be expanded to other areas.

## 4 Experimental Section

### Materials and Reagents

3,3′,5,5′‐tetramethylbenzidine (TMB) was purchased from American Acros reagent. Bovine Serum Protein (BSA) and N‐hydroxysuccinimide (NHS) were bought from Sigma–Aldrich. 1‐(3‐dimethylaminopropyl)‐3‐ethylcarbodiimide hydrochloride (EDC) and 5,5‐dimethyl‐1‐pyrrolin‐N‐oxide were obtained from Shanghai Aladdin Biochemical Technology Co., LTD. Fumaric acid, Ce(NO_3_)_3_·6H_2_O and citric acid were purchased from Shanghai Maclin Biochemical Technology Co., LTD. FeCl_3_·6H_2_O, N,N‐dimethylformamide (DMF), hexamine (HMTA), ethanediamine, H_2_O_2_, dimethylsulfoxide, CH_3_COOH, CH_3_COONa and ethanol were obtained from Sinopharm Group Chemical reagent Co., LTD. The Staphylococcus aureus enterotoxin B (SEB) and Anti‐SEB antibody Hm4 were provided by Chongqing Third Military Medical University.

### Apparatus

The transmission electron micrographs (TEM) were acquired from a Philips Tecnai‐12 microscope (Holland) at 120 kV accelerating voltage. Hitachi S‐4800 scanning electron microscope (Japan) was exploited to obtain the scanning electron micrographs (SEM) at 15 kV accelerating voltage. High‐resolution transmission electron micrographs (HRTEMs) were acquired using an FEI Tecnai G2 F30 S‐TWIN field‐emission transmission electron microscope (Field Electron and Ion Co. [FEI], Hillsboro, Oregon) at an acceleration voltage of 300 kV. X‐ray photoelectron spectra (XPS) were acquired using an ESCALAB 250Xi spectrometer. The D8 advanced X‐ray diffractometer (Bruker Co., Germany) was used for investigating X‐ray diffraction (XRD) patterns at room‐temperature. Zeta potential experiments were obtained with a ZEN3690 nanometer particle size analyzer (Malvern Instruments LTD., UK). Ultraviolet‐visible (UV–vis) experiments were completed with a UV‐2500 spectrophotometer (Beijing General General Instrument Co., Ltd). Fluorescence (FL) experiments were performed with the F‐7000 spectrophotometer (Hitachi LTD., Japan). The enhancement mechanism of the activity of the OXD‐like was investigated using a UV–Vis‐near‐infrared absorption spectrometer (Varian, America) and an electrochemical workstation (Shanghai Chenhua Instrument Co., LTD). All pH values were measured via S‐25 digital pH‐meter and glass composite electrode.

### Synthesis of Fe‐88A, Fe‐88A@CeO_2_, and Fe‐88A@CeO_2_/CDs Nanozymes

Fe‐88A was first prepared according to previous literature.^[^ ^57^ ^]^ Fumaric acid (1.16 g) and FeCl_3_·6H_2_O (1.62 g) were dissolved in 100 mL of N,N‐dimethylformamide (DMF), followed by transferring to an oil bath at 100 °C for condensation and reflux of 2 h. Subsequently, the resulting yellow product was collected through centrifugation, washed twice with DMF, and dried overnight under vacuum conditions at 60 °C.

Fe‐88A@CeO_2_ was prepared according to the reported work with modifications.^[^ ^46^ ^]^ 0.1 g of Fe‐88A was dispersed in a mixture of 40 mL deionized water and 40 mL anhydrous ethanol, followed by ultrasonic dissolution. The resulting solution was then placed in an oil bath at 70 °C and subjected to magnetic stirring. Subsequently, 0.15 g of Ce(NO_3_)_3_·6H_2_O and 0.4 g of C_6_H_12_N_4_ were sequentially added for refluxing for 2 h, and the reaction mixture was cooled to room‐temperature. The obtained product was washed alternately three times with anhydrous ethanol and deionized water before being vacuum dried at 60 °C. For comparative experiments, the total mass of Ce(NO_3_)_3_·6H_2_O was 0.5, 0.1, and 0.2 g, respectively, while keeping the other experimental conditions unchanged.

Fe‐88A@CeO_2_/CDs nanozyme was prepared by adding Ce(NO_3_)_3_·6H_2_O and C_6_H_12_N_4_ with 10 mg CDs during the preparation of Fe‐88A@CeO_2_, followed by refluxing for 2 h. The products were then cooled to room‐temperature, centrifuged, collected, and washed alternately with anhydrous ethanol and deionized water three times, and dried under vacuum at 60 °C. For comparison experiments, the total mass of CDs was set as 1, 5 and 15 mg, respectively, while keeping the other experimental conditions unchanged.

### Synthesis of Fe‐88A@CeO_2_@CDs (adsorption) Nanozymes:

The synthesized Fe‐88A@CeO_2_ was dispersed via ultrasonic treatment in a mixed solution containing 40 mL deionized water and 40 mL anhydrous ethanol. Subsequently, 10 mg of CDs were added, and the mixture was refluxed for 2 h. The resulting product was then collected by centrifugation, washed alternately three times with anhydrous ethanol and deionized water, and finally dried in vacuum at 60 °C.

### Synthesis of CDs

A mixture containing 4.8 g of citric acid and 1.675 mL of ethylenediamine was dispersed in 50 mL of deionized water. The resulting solution was subjected to ultrasonic dissolution to ensure uniformity before being transferred into a Teflon‐lined reactor with a capacity of 100 mL. The reaction took place at a temperature of 200 °C for a duration of 6 h, followed by cooling the suspension to room‐temperature and subsequent dialysis treatment. Finally, freeze‐drying was performed to obtain a solid powder form of CDs.

### Synthesis of CeO_2_

The mixture of 0.15 g Ce(NO_3_)_3_·6H_2_O and 0.4 g C_6_H_12_N_4_ was sequentially added to a solution containing 40 mL deionized water and 40 mL anhydrous ethanol. After ultrasonic dissolution, the resulting solution was stirred at room‐temperature for 30 min, followed by reflux in an oil bath at 70 °C for 2 h. Subsequently, it was cooled to room‐temperature, centrifuged, and collected. The obtained product was washed alternately with anhydrous ethanol and deionized water three times before being dried under vacuum conditions at 60 °C.

### Preparation of Fe‐88A@CeO_2_/CDs@Hm4

To obtain SEB antibody (Hm4)‐modified Fe‐88A@CeO_2_/CDs, 10 µL EDC (1 mg mL^−1^) and 10 µL NHS (1 mg mL^−1^) were added to a solution of 4 mL Fe‐88A@CeO_2_/CDs (1 mg mL^−1^), followed by the addition of 20 µL Hm4 (1 mg mL^−1^) and incubation at 4 °C for 2 h and overnight. Afterward, 5.0 µL of BSA was introduced, and the probe was collected via low‐temperature centrifugation, resuspended in PBS (pH 7.4), and stored at 4 °C for subsequent use.

### Oxidase‐Like Activity and Enzyme Kinetics Analysis

The OXD‐like activity of Fe‐88A@CeO_2_/CDs was investigated using 3,3′,5,5′‐tetramethylbenzidine (TMB) as a chromogenic agent. A mixture of 100 µL Fe‐88A@CeO_2_/CDs (1 mg mL^−1^) and 50 µL TMB (20 mm) was added to an acetic acid buffer solution (0.1 m, pH 4.5). After incubation at 30 °C for 5 min, the mixture was taken to measure the changes of the absorption peak at 652 nm. The steady‐state kinetics analysis of the Fe‐88A@CeO_2_/CDs‐TMB system was conducted by measuring the absorbance at 652 nm. The kinetic parameters were determined using the Michaelis–Menten equation:

(8) $$\frac{1}{v}=\frac{1}{\left[S\right]}\frac{K_{m}}{V_{\max}}+\frac{1}{V_{\max}}$$

where *v* represents the initial velocity, [*S*] denotes the substrate concentration, *V*_max_ indicates the maximum reaction velocity, and *K*_m_ represents the Michaelis–Menten constant.

### Colorimetric‐Fluorescence Bimodal Detection of SEB

First, 50 µL of Staphylococcal enterotoxin B (SEB) with varying concentrations was added to a 100 µL probe solution and incubated at room‐temperature for 15 min. Subsequently, 100 µL immune complex was introduced into an acetic acid buffer solution (0.1 m, pH 4.5), followed by the addition of 50 µL of TMB solution (20 mm). The UV–vis absorption spectra and fluorescence spectra of the reaction mixture were subsequently recorded after a reaction time of 5 min.

### Determination of Real Samples

To evaluate the practicality of the proposed method in real samples detection, milk samples and water samples from the school supermarket were selected for recovery experiments. The commercial skimmed milk samples were treated with acetic acid solution and centrifuged at 5000 r min^−1^ for 10 min. The resulting supernatant was then diluted 100‐fold with ultrapure water. The treated water and milk samples were spiked with standard analyte solutions, and the recovery rates were calculated.

## Conflict of Interest

The authors declare no conflict of interest.

## Supporting information



Supporting Information

## Data Availability

The data that support the findings of this study are available in the supplementary material of this article.
