# Supplementary material for: Three‐Channel Electron Engineered Fe‐88A@CeO2/CDs Nanozyme with Enhanced Oxidase‐Like Activity for Efficient Biomimetic Catalysis
Source: Adv Sci (Weinh). 2025 Aug 31;12(42):e09713. doi: 10.1002/advs.202509713 (PMC12622548; doi:10.1002/advs.202509713)
Supplement: Supplementary file 1 — Supporting Information [file ADVS-12-e09713-s001.docx]

Supporting Information

Three-Channel Electron Engineered Fe-88A@CeO_2_/CDs Nanozyme with Enhanced Oxidase-Like Activity for Efficient Biomimetic Catalysis

Kai Liu ^a1^, Haibing Zhu ^a1^, Feng Shi ^a1^, Juan Li ^a^*, Xiang Li ^a^, Zijun Lai ^a^, Haibo Li ^b^, Hao Zeng ^b^*, Zhanjun Yang ^a^* and Huan Pang ^a^*


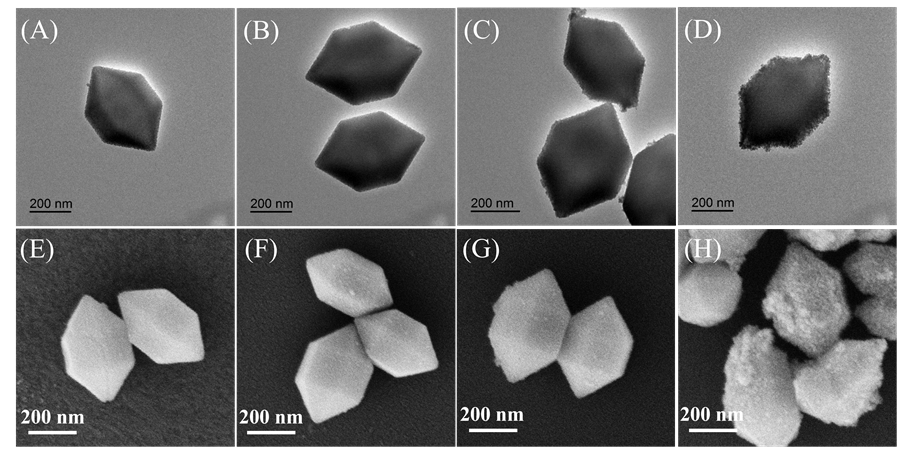


**Figure S1.** (A-D) TEM images of Fe-88A@CeO_2_-0.05, Fe-88A@CeO_2_-0.1, Fe-88A@CeO_2_-0.15 and Fe-88A@CeO_2_-0.2; (E-H) SEM images of Fe-88A@CeO_2_-0.05, Fe-88A@CeO_2_-0.1, Fe-88A@CeO_2_-0.15 and Fe-88A@CeO_2_-0.2.


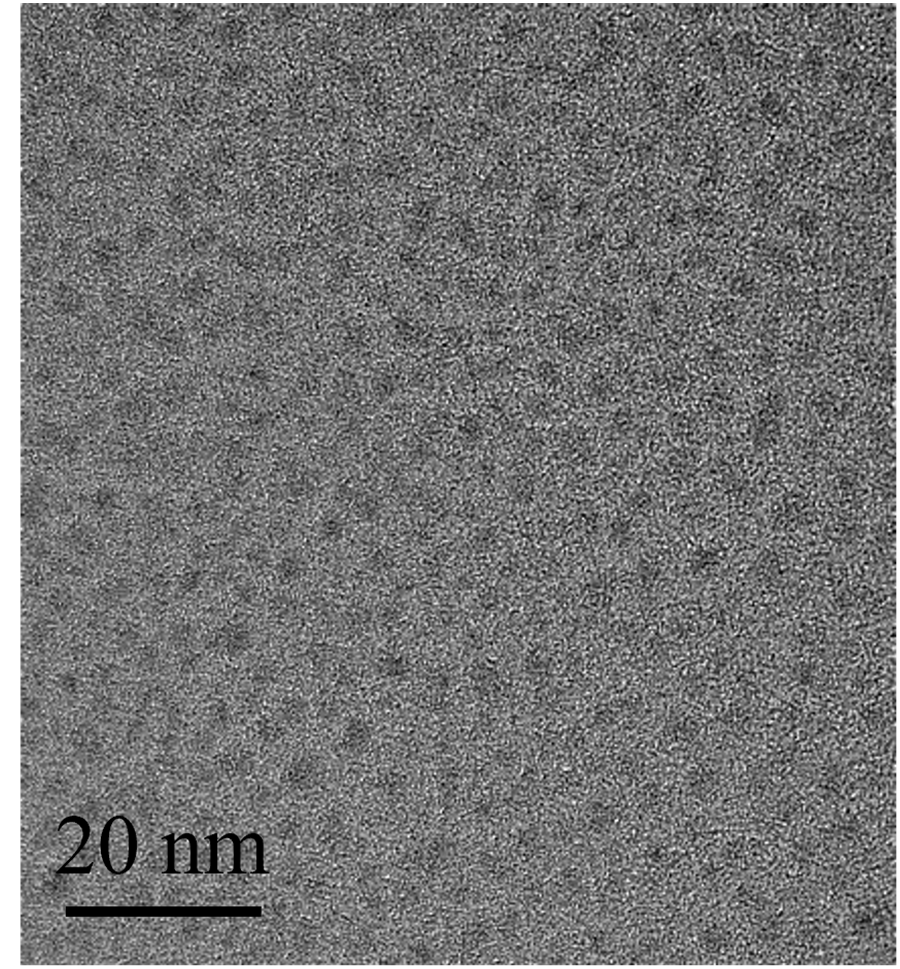


**Figure S2.** TEM image of CDs.


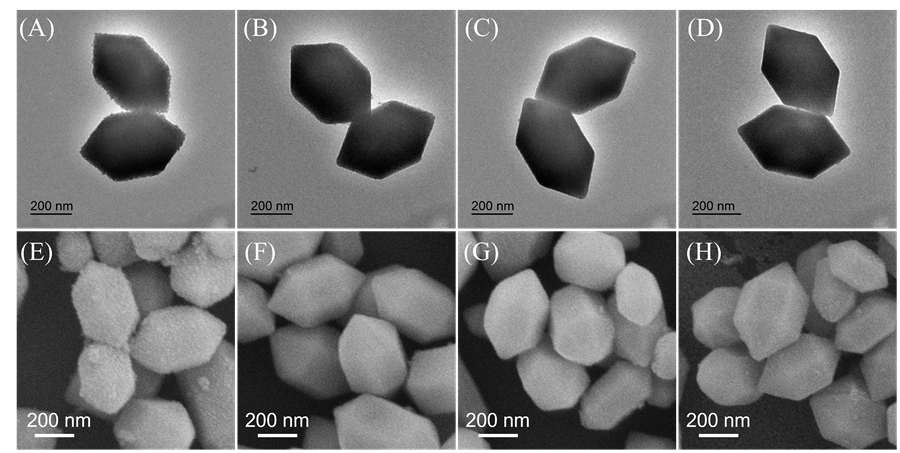


**Figure S3.** (A-D) TEM images of Fe-88A@CeO_2_/CDs-1, Fe-88A@CeO_2_/CDs-5, Fe-88A@CeO_2_/CDs-10 and Fe-88A@CeO_2_/CDs-15; (E-H) SEM images of Fe-88A@CeO_2_/CDs-1, Fe-88A@CeO_2_/CDs-5, Fe-88A@CeO_2_/CDs-10 and Fe-88A@CeO_2_/CDs-15.


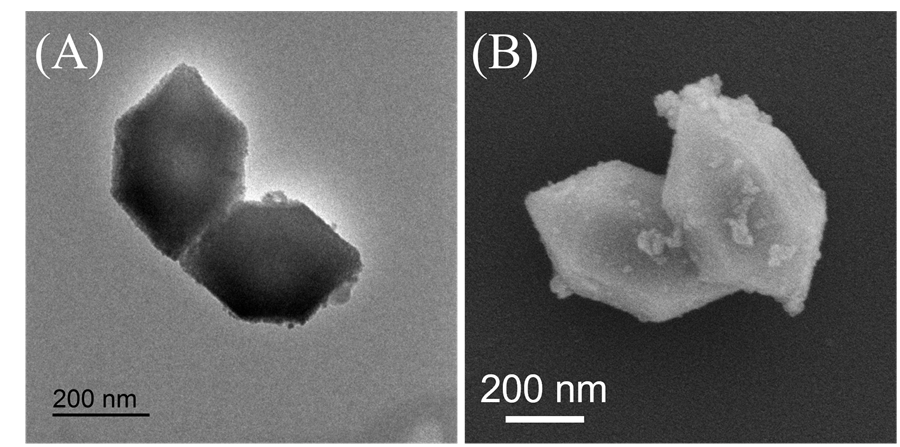


**Figure S4.** (A) TEM and (B) SEM images of Fe-88A@CeO_2_@CDs (adsorption).

As shown in **Figures S5A and S5B**, the line scan profiles of Fe-88A@CeO_2_/CDs (in-situ) and Fe-88A@CeO_2_@CDs (adsorption) were studied. In the case of introducing exogenous carbon dots with the same mass, the counts of C (red line) in Fe-88A@CeO_2_/CDs (in-situ) gradually decreases along the line scan path. This can be attributed to that the CDs was coated by CeO_2_ in the in-situ formation process of CeO_2_, as well as the subsequent embedding of CeO_2_/CDs into the pores of Fe-88A. Thereby, only a small portion unavoidably appears outside. In contrast, the overall C signal in Fe-88A@CeO_2_@CDs (adsorption) is higher than that in Fe-88A@CeO_2_/CDs (in-situ), suggesting that a large number of CDs were enriched on the exterior of Fe-88A@CeO_2_ rather than the pores of Fe-88A during the adsorption process, which is highly consistent with our expectations. Additionally, in the inner region (corresponding to the middle segment of the line scan) of Fe-88A@CeO_2_@CDs (adsorption), the counts of O (yellow line) and Ce (blue line) are significantly lower than the C counts. These results further confirm the in-situ incorporation of CDs into Fe-88A pores.


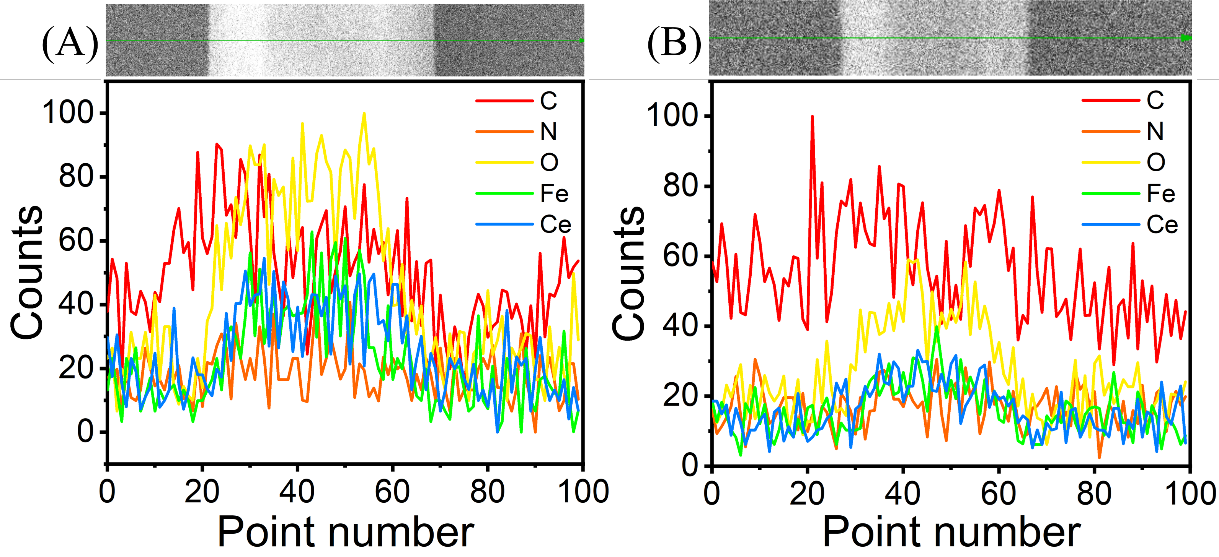


**Figure S5.** Line scan images of the nanozyme structures: (A) Fe-88A@CeO_2_/CDs; (B) Fe-88A@CeO_2_@CDs (adsorption).


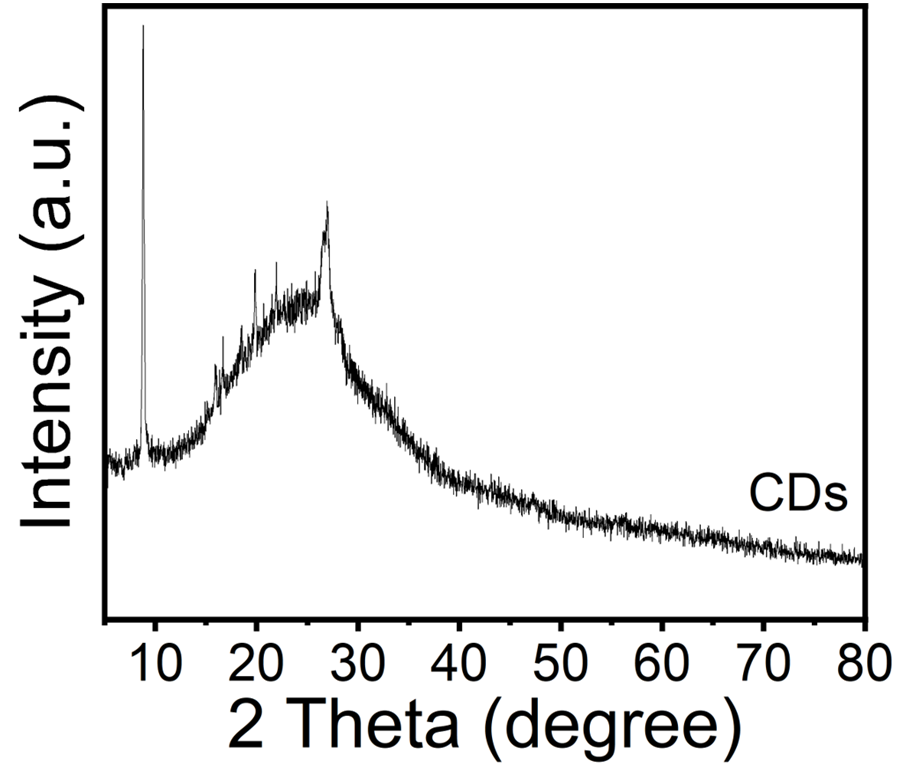


**Figure S6.** (B) XRD pattern of CDs.


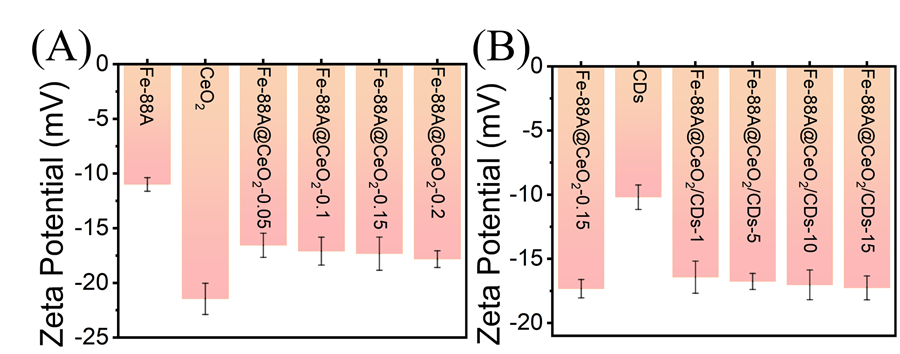


**Figure S7.** (A) Zeta potential of Fe-88A@CeO_2_ regulated with Ce(NO_3_)_3_·6H_2_O; (B) Zeta potential Fe-88A@CeO_2_/CDs regulated with CDs.


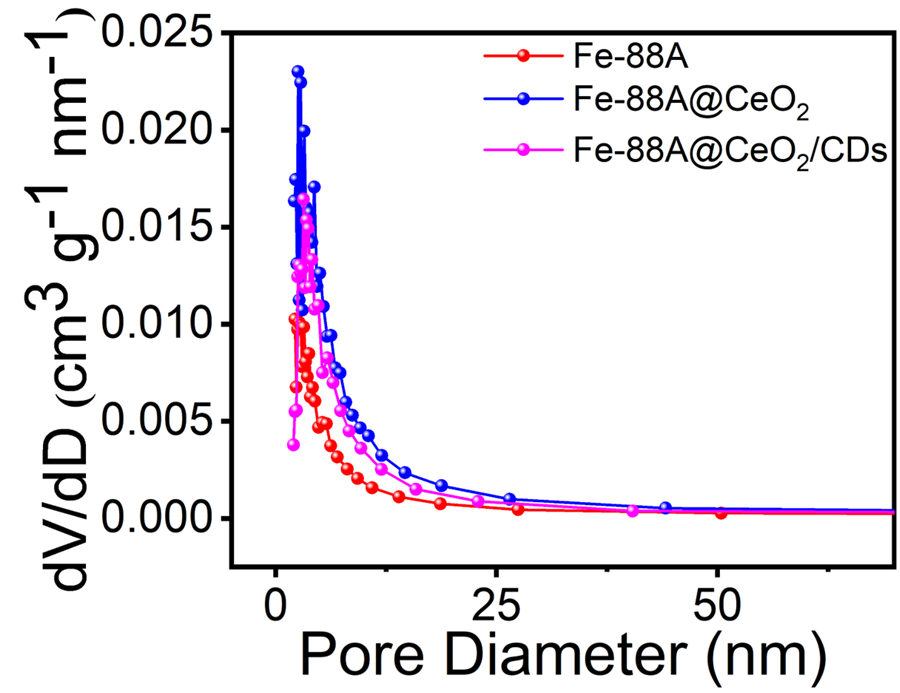


**Figure S8.** Pore size distribution of Fe-88A, Fe-88A@CeO_2_, and Fe-88A@CeO_2_/CDs.

**Table S1.** BET specific surface area (S_BET_), total volume (V) and pore size distribution (D) of the synthesized nanozymes

| Samples | S_BET_ (m^2^ g^-1^) | V (cm^3^ g^-1^) | D (nm) |
| --- | --- | --- | --- |
| Fe-88A | 42.04 | 0.09 | 5.03 |
| Fe-88A@CeO_2_ | 99.96 | 0.14 | 9.97 |
| Fe-88A@CeO_2_/CDs | 64.56 | 0.09 | 14.37 |


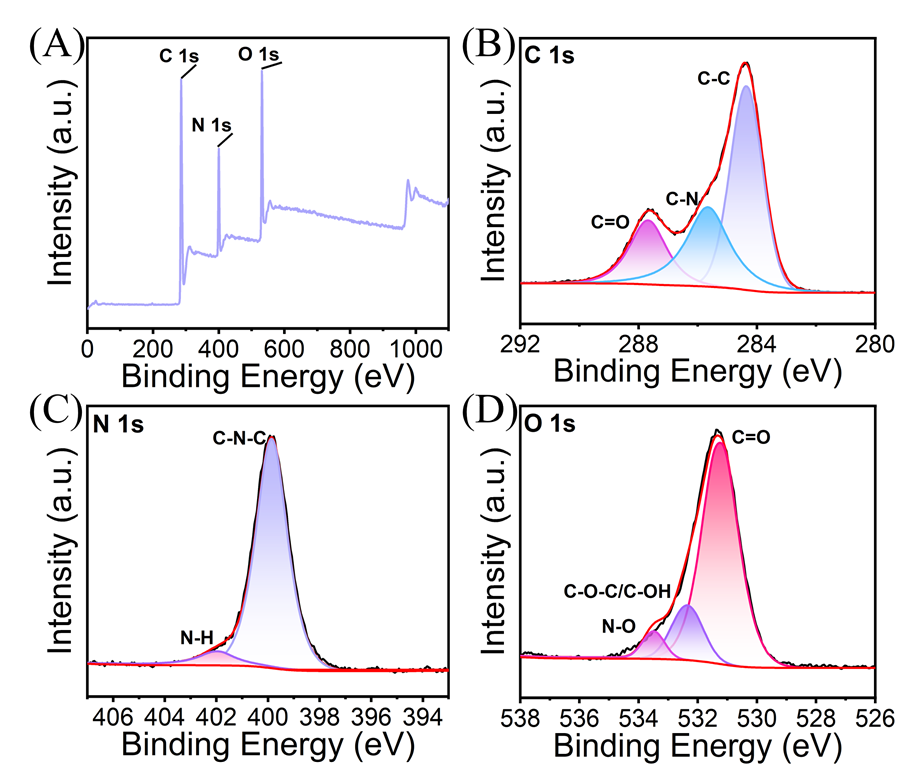


**Figure S9.** XPS spectra of CDs: (A) Full XPS spectrum and high-resolution XPS spectra of (B) C 1s, (C) N 1s and (D) O 1s.


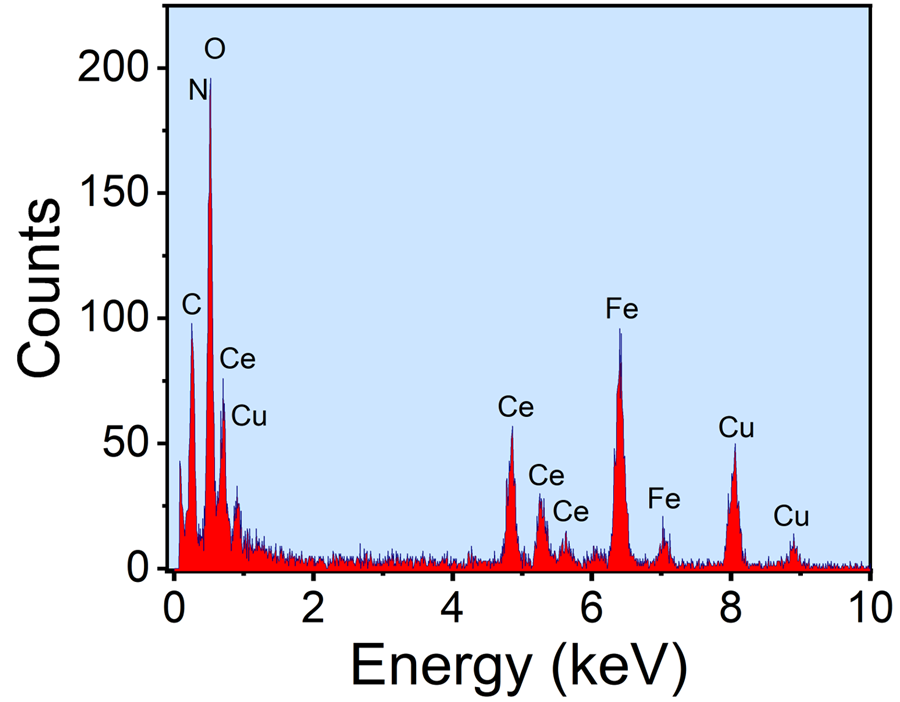


**Figure S10.** EDS of Fe-88A@CeO_2_/CDs.


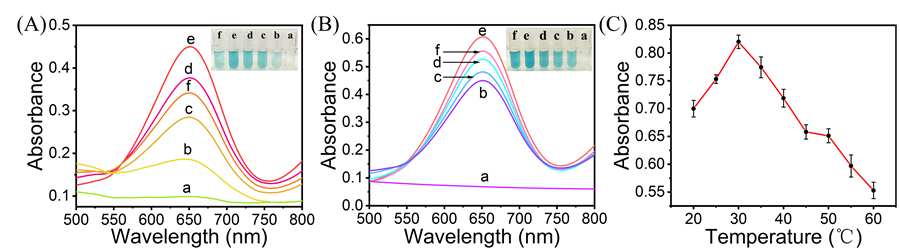


**Figure S11.** (A) Oxidase-like activity of Ce(NO_3_)_3_·6H_2_O-regulated nanozymes (a: CeO_2_+TMB, b: Fe-88A+TMB, c: Fe-88A@CeO_2_-0.05 d: Fe-88A@CeO_2_-0.1+TMB, e: Fe-88A@CeO_2_-0.15+TMB, f: Fe-88A@CeO_2_-0.2+TMB); (B) Oxidase-like activity of CDs-regulated nanozymes (a: CDs+TMB, b: Fe-88A@CeO_2_-0.15, c: Fe-88A@CeO_2_/CDs-1 d: Fe-88A@CeO_2_/CDs-5+TMB, e: Fe-88A@CeO_2_/CDs-10+TMB, f: Fe-88A@CeO_2_/CDs-15+TMB). (C) Temperature optimization of the enzymatic reaction.


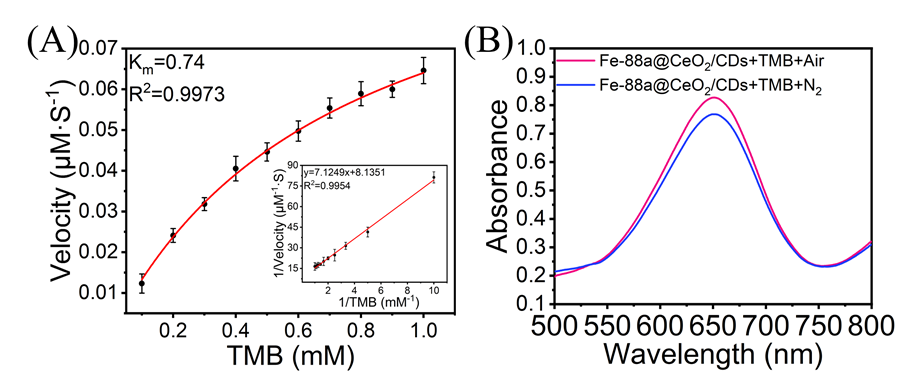


**Figure S12.** (A) The kinetics curves of Fe-88A@CeO_2_ with TMB as substrate, and the inset shows the corresponding double reciprocal plots of Fe-88A@CeO_2_/CDs; (B) Oxidase-like activity of Fe-88A@CeO_2_/CDs+TMB system in air atmosphere and aerating N_2_ to remove partially dissolved O_2_.

**Table S2.** Comparison of steady-state kinetic parameters of Fe-88A@CeO_2_, Fe-88A@CeO_2_/CDs, HRP and other nanozyme materials.

| **Enzymes/Nanozymes** | **TMB** | | **Ref.** |
| --- | --- | --- | --- |
|  | *K_m_* (mM) | *V_max_* (10^-8^ Ms^-1^) |  |
| **Horseradish peroxidase** | 0.43 | 10.0 | [S1] |
| **CoFeCe three-atom hydroxide** | 4.47 | 6.93 | [S2] |
| **CeO_2_** | 10.99 | 6.98 | [S3] |
| **Fe-MOF** | 2.6 | 5.6 | [S4] |
| **ZIF-67 nanosheets** | 13.69 | 0.35 | [S5] |
| **CeO_2_/NH_2_-MIL-88B(Fe)** | 0.86 | 1.10 | [S6] |
| **Fe-CDs** | 0.72 | 18.13 | [S7] |
| **MIL-101** | 6.71 | 60.10 | [S8] |
| **Fe-88A@CeO_2_** | 0.74 | 11.12 | This work |
| **Fe-88A@CeO_2_/CDs** | 0.69 | 24.28 |  |


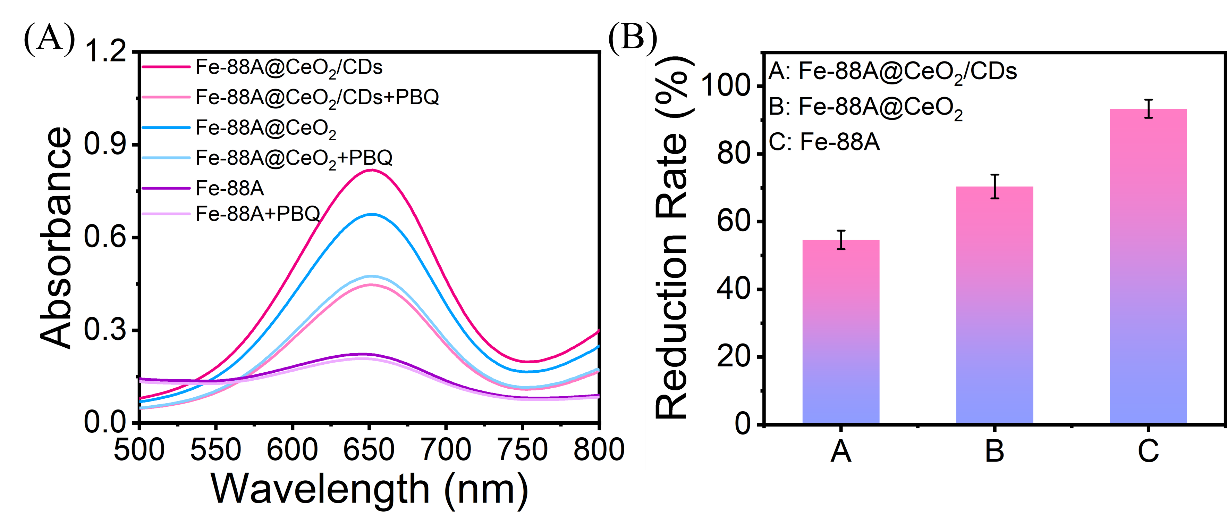


**Figure S13.** (A) The effect of p-benzoquinone radical scavenger on the absorbance at 652 nm of Fe-88A@CeO_2_/CDs+TMB, Fe-88A@CeO_2_+TMB, and Fe-88A+TMB systems; (B) The effect of p-benzoquinone radical scavenger on the catalytic activity reduction rate of Fe-88A@CeO_2_/CDs, Fe-88A@CeO_2_, and Fe-88A.


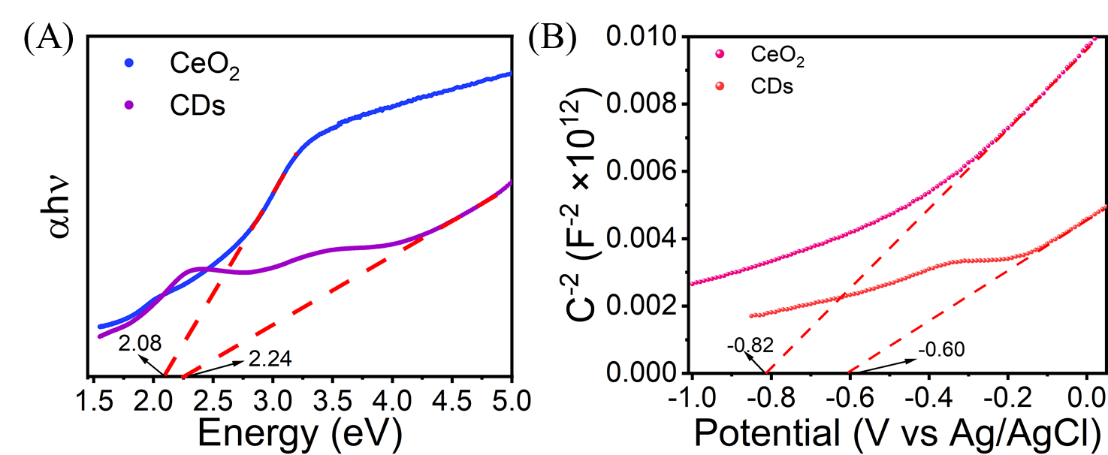


**Figure S14.** (A) Tauc plots of CDs and CeO_2_; (B) Mott-Schottky plots and the potential is vs. Ag/AgCl for CDs and CeO_2_.


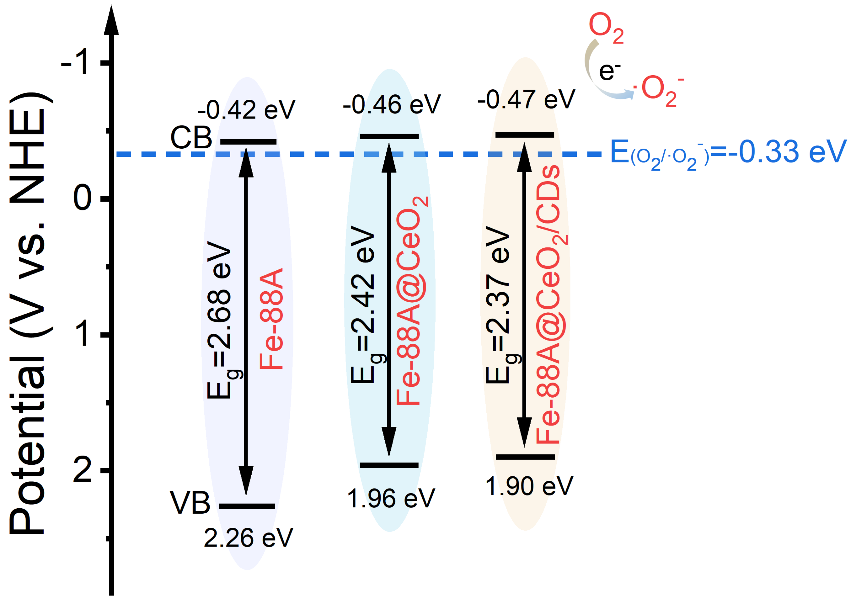


**Figure S15.** Schematic illustration of the band structures and oxygen reduction reaction (ORR) activity of Fe-88A, Fe-88A@CeO_2_, and Fe-88A@CeO_2_/CDs.


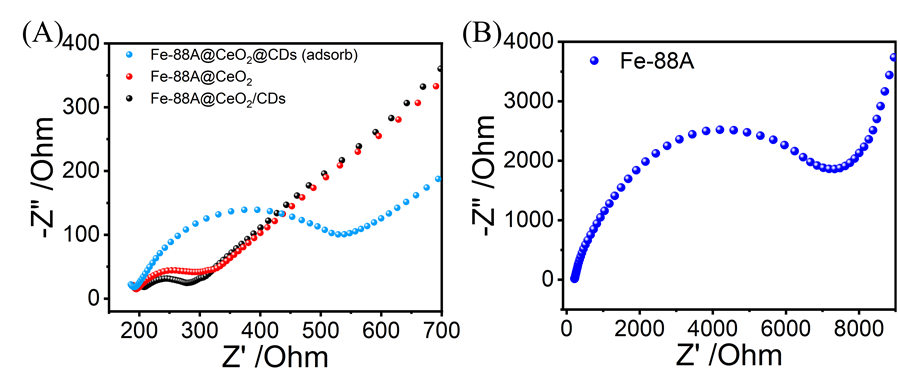


**Figure S16.** (A) The Nyquist curves of Fe-88A@CeO_2_, Fe-88A@CeO_2_/CDs and Fe-88A@CeO_2_@CDs (adsorption); (B) The Nyquist curves of Fe-88A.


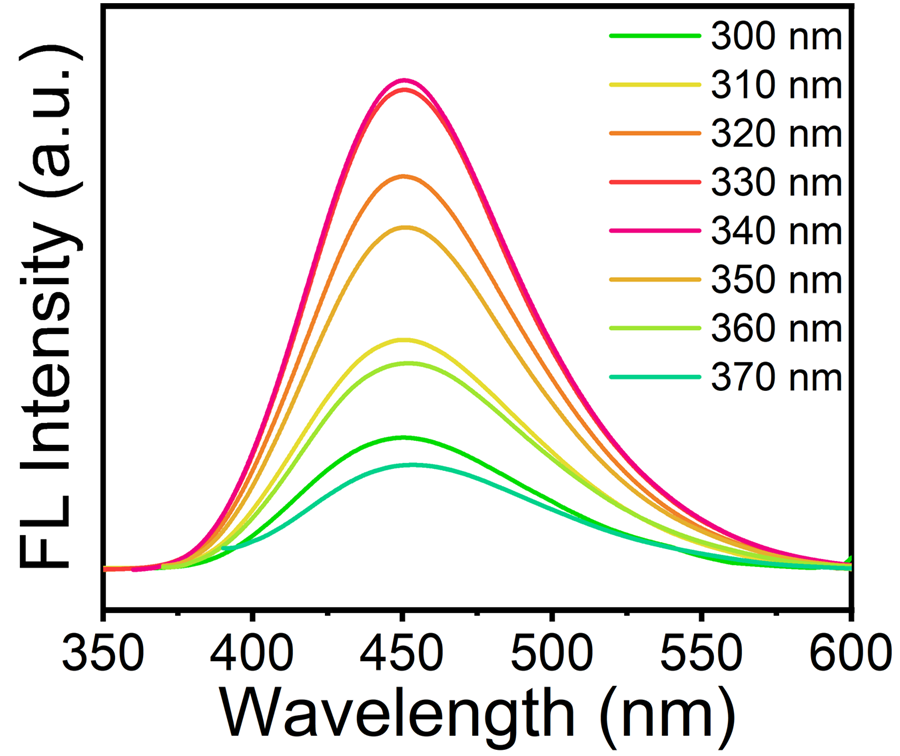


**Figure S17.** Fluorescence emission spectra of Fe-88A@CeO_2_/CDs under different excitations.


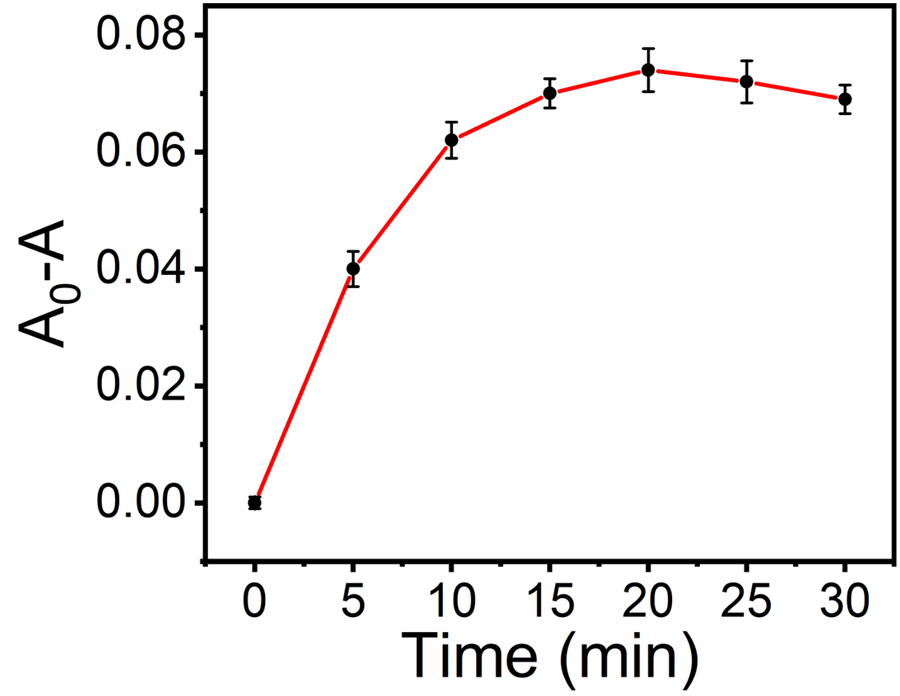


**Figure S18.** Optimization of incubation time for detection of SEB.


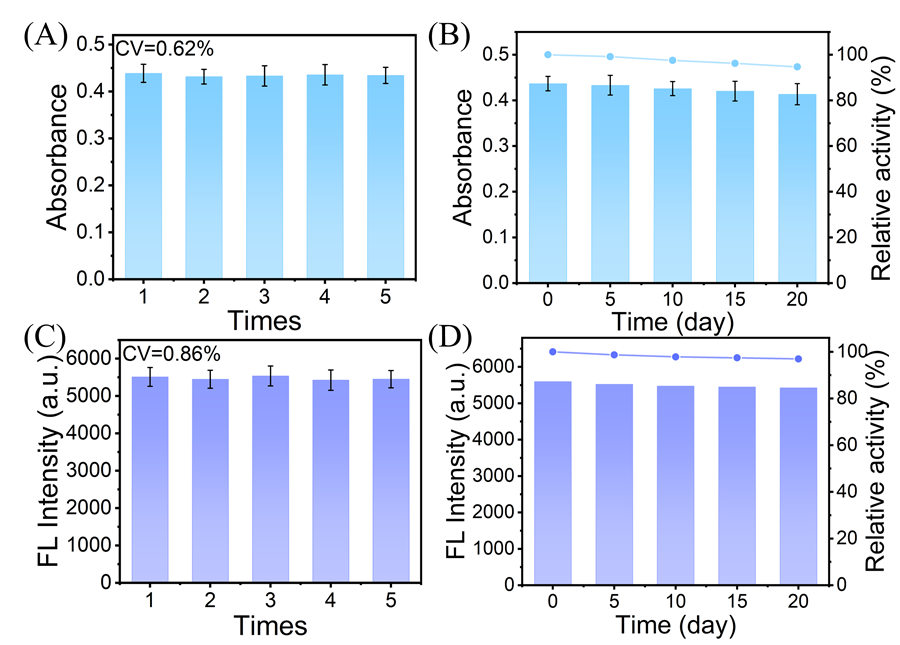


**Figure S19.** Reproducibility and stability of colorimetric fluorescence bimodal immunosensor.

**Table S3.** Comparison of the analytical performance of Fe-88A@CeO_2_/CDs with other SEB sensors.

| **Methods** | **System** | **Linear range**  **(ng/mL)** | **LOD (ng/mL)** | | **Ref.** |
| --- | --- | --- | --- | --- | --- |
| Fluorescence | J-cf-HBN@mAbs (Anti-SEB) | 1.56-400 | 1.56 | [S9] | |
| Colorimetry |  | 0.19-400 | 0.09 |  |  |
| Photothermal | Nb7@CPNs (Anti-SEB) | 1-256 | 0.58 | [S10] | |
| Colorimetry |  | 2-64 | 1.68 |  |  |
| Colorimetry | HAg/Au NSts-LFIA (Anti-SEB) | 0.19-100 | 0.29 | [S11] | |
| Photothermal |  | 0.19-100 | 0.09 |  |  |
| Fluorescence | RANbody-ELISA (Anti-SEB) | 0.31-2500 | 0.12 | [S12] | |
| Colorimetry |  | 0.31-2500 | 0.24 |  |  |
| Colorimetry | Fe-88A@CeO_2_/CDs@Hm4 (Anti-SEB) | 0.008-200 | 0.003 | This work | |
| Fluorescence |  | 0.005-200 | 0.001 |  |  |

**Table S4** Recoveries of SEB spiked in milk and water samples (n=3).

| **Method** | **Samples** | **Added (ng/mL)** | **Detected (ng/mL)** | **Recovery (%)** | **RSD (%)** |
| --- | --- | --- | --- | --- | --- |
| **Colorimetric** | Milk | 5 | 4.94 | 98.80 | 0.58 |
|  |  | 30 | 30.24 | 100.80 | 0.41 |
|  |  | 100 | 103.49 | 103.49 | 0.79 |
|  | Water | 5 | 5.04 | 100.80 | 1.41 |
|  |  | 30 | 30.81 | 102.70 | 0.36 |
|  |  | 100 | 94.83 | 94.83 | 1.58 |
| **Fluorometric** | Milk | 0.1 | 0.11 | 110.00 | 1.29 |
|  |  | 10 | 9.96 | 99.34 | 0.33 |
|  |  | 50 | 50.41 | 100.82 | 0.12 |
|  | Water | 0.1 | 0.095 | 95.00 | 0.76 |
|  |  | 10 | 10.22 | 102.20 | 0.42 |
|  |  | 50 | 49.47 | 98.84 | 0.23 |

**Reference**

1. Gao, L. Z.; Zhuang, J.; Zhang, J. B.; Zhang, Y.; Gu, N.; Wang, T. H.; Feng, J.; Yang, D. L.; Perrett, S.; Yan, X. Y. Intrinsic Peroxidase-Like Activity of Ferromagnetic Nanoparticles. *Nat. Nanotechnol.* **2007**, 2, 577-583.
2. Shang, H. Y.; Zhang, X. F.; Ding, M. L.; Zhang, A. P.; Wang. C. A Smartphone-Assisted Colorimetric and Photothermal Probe for Glutathione Detection Based on Enhanced Oxidase-Mimic CoFeCe Three-atom Nanozyme in Food. *Food Chem.* **2023**, 423, 136296.
3. Patel, V.; Jose, L.; Philippot, G.; Aymonier, C.; Ineerbaev, T.; McCourt, L. R.; Ruppert, M. G.; Qi, D. C.; Li, W.; Qu, J. T.; Zheng, R. K.; Cairney, J.; Yi, J. B.; Vinu, A.; Karakoti, A. S. Fluoride-Assisted Detection of Glutathione by Surface Ce^3+^/Ce^4+^ Engineered Nanoceria. *: J. Mater. Chem. B,* **2022***,*10, 9855.
4. Xu, W. Q.; Jiao, L.; Yan, H. Y.; Wu, Y.; Chen, L. J.; Gu, W. L.; Du, D.; Lin, Y. H.; Zhu, C. Z. Glucose Oxidase-Integrated Metal-Organic Framework Hybrids as Biomimetic Cascade Nanozymes for Ultrasensitive Glucose Biosensing. *ACS Appl. Mater. Interfaces* **2019**, 11, 22096-22101.
5. Wang, S. J.; Xu, D. P.; Ma, L.; Qiu, J. X.; Wang, X.; Dong, Q. L.; Zhang, Q.; Pan, J.; Liu, Q. Ultrathin ZIF-67 Nanosheets as A Colorimetric Biosensing Platform for Peroxidase-Like Catalysis. *Anal. Bioanal. Chem.* **2018**, 410, 7145-7152.
6. Li, J. Z.; Tang, X. H.; Guo, X. J.; Han, W.; Wang, S. K.; Zha, F.; Tian, H. F.; Chang, Y. CeO_2_/NH_2_-MIL-88B(Fe) Composites with Peroxidase-Like Activity for Colorimetric Detection and Photo-Enzymatic Synergetic Degradation of Ciprofloxacin Hydrochloride. *J. Environ. Chem. Eng.* **2024**, 12, 114734.
7. Zhang, R. L.; Liu, L.; Li, W.; Luo, X. G.; Wu, F. S. Luminescent Carbon Dots with Excellent Peroxidase Mimicking Property for Fluorometric and Colorimetric Detection of Glucose. *Colloids Surf. B Biointerfaces* **2023**, 222, 113125.
8. Xu, W. Q.; Kang, Y. K.; Jiao, L.; Wu, Y.; Yan, H. Y.; Li, J. L.; Gu, W. L.; Song, W. Y.; Zhu, C. Z. Tuning Atomically Dispersed Fe Sites in Metal-Organic Frameworks Boosts Peroxidase-Like Activity for Sensitive Biosensing. *Nano-Micro Lett.* **2020**, 12, 184.
9. Shen, X. A.; Zhou, H. X.; Chen, X. R.; Wu, J. Y.; Wu, J. Y.; Su, Y.; Huang, X. L.; Xiong, Y. H. Janus Plasmonic-aggregation Induced Emission Nanobeads as High-Performance Colorimetric-Fluorescent Probe of Immunochromatographic Assay for the Ultrasensitive Detection of Staphylococcal Enterotoxin B in Milk. *Biosens. Bioelectron.* **2024**, 261, 116458.
10. Wu, H. F.; Li. Y. H.; Li, Y. C.; Cui, Y.; Jia, C. H.; Wang, J. L.; Pan, J. C.; Yu, G. G.; Zhang, X. L.; Wang, X. T.; Guo, P. R.; Ji, Y. W. The “Umbrella of Tolerance”: Nanobodies-Armed Photothermal Lateral Flow Immunoassay for the Detection of Staphylococcal Enterotoxin B. *Chem. Eng. J.* **2023**, 470, 144273.
11. Wang, X. T.; Chang, S. H.; Zhang, X. L.; Guo, X. H.; Xu, Y. J.; Yang, D.; Luo, L. P.; Wang, J. M.; Wu, H. F.; Cui, Y.; Wang, J. L.; Ji, Y. W. A Highly Sensitive Dual-Mode Lateral Flow Immunoassay Based on Plasmonic Hollow Ag/Au Nanostars Enhancing Light Absorption. *Talanta* **2024**, 280, 126683.
12. Zhang, Y.; Li, Y. H.; Li, M.; Tian, Y. D.; Zhou, T.; Yu, Y.; Zheng, W. X. A Bifunctional Protein RANbody Based on Nanobody Facilitates Dual-Mode Immunoassay of Staphylococcal Enterotoxin B in Food Samples. *Sens. Actuators B Chem.* **2024**, 418, 136295.
